# Assessing the causal effect of genetically predicted metabolites and metabolic pathways on stroke

**DOI:** 10.1186/s12967-023-04677-4

**Published:** 2023-11-17

**Authors:** Tianlong Zhang, Yina Cao, Jianqiang Zhao, Jiali Yao, Gang Liu

**Affiliations:** 1 Department of Critical Medicine, The Fourth Affiliated Hospital of Zhejiang University School of Medicine, Yiwu, Zhejiang China; 2 Department of Neurology, The Fourth Affiliated Hospital of Zhejiang University School of Medicine, Yiwu, Zhejiang China; 3 Department of Cardiology, The Fourth Affiliated Hospital of Zhejiang University School of Medicine, Yiwu, Zhejiang China; 4 Department of Critical Care Medicine, Jinhua Hospital Affiliated to Zhejiang University, Jinhua, Zhejiang China; 5 Department of Infection Control, The Fourth Affiliated Hospital of Zhejiang University School of Medicine, Yiwu, Zhejiang China

**Keywords:** Metabolites, Stroke, Ischemic stroke, Large artery stroke, Cardioembolic stroke, Small vessel stroke, Intracerebral hemorrhage, Subarachnoid hemorrhage, Transient ischemic attack, Genome-wide association studies, Mendelian randomization

## Abstract

**Background:**

Stroke is a common neurological disorder that disproportionately affects middle-aged and elderly individuals, leading to significant disability and mortality. Recently, human blood metabolites have been discovered to be useful in unraveling the underlying biological mechanisms of neurological disorders. Therefore, we aimed to evaluate the causal relationship between human blood metabolites and susceptibility to stroke.

**Methods:**

Summary data from genome-wide association studies (GWASs) of serum metabolites and stroke and its subtypes were obtained separately. A total of 486 serum metabolites were used as the exposure. Simultaneously, 11 different stroke phenotypes were set as the outcomes, including any stroke (AS), any ischemic stroke (AIS), large artery stroke (LAS), cardioembolic stroke (CES), small vessel stroke (SVS), lacunar stroke (LS), white matter hyperintensities (WMH), intracerebral hemorrhage (ICH), subarachnoid hemorrhage (SAH), transient ischemic attack (TIA), and brain microbleeds (BMB). A two‐sample Mendelian randomization (MR) study was conducted to investigate the causal effects of serum metabolites on stroke and its subtypes. The inverse variance-weighted MR analyses were conducted as causal estimates, accompanied by a series of sensitivity analyses to evaluate the robustness of the results. Furthermore, a reverse MR analysis was conducted to assess the potential for reverse causation. Additionally, metabolic pathway analysis was performed using the web-based MetOrigin**.**

**Results:**

After correcting for the false discovery rate (FDR), MR analysis results revealed remarkable causative associations with 25 metabolites. Further sensitivity analyses confirmed that only four causative associations involving three specific metabolites passed all sensitivity tests, namely ADpSGEGDFXAEGGGVR* for AS (OR: 1.599, 95% CI 1.283–1.993, p = 2.92 × 10^−5^) and AIS (OR: 1.776, 95% CI 1.380–2.285, p = 8.05 × 10^−6^), 1-linoleoylglycerophosph-oethanolamine* for LAS (OR: 0.198, 95% CI 0.091–0.428, p = 3.92 × 10^−5^), and gamma-glutamylmethionine* for SAH (OR: 3.251, 95% CI 1.876–5.635, p = 2.66 × 10^−5^), thereby demonstrating a high degree of stability. Moreover, eight causative associations involving seven other metabolites passed both sensitivity tests and were considered robust. The association result of one metabolite (glutamate for LAS) was considered non-robust. As for the remaining metabolites, we speculate that they may potentially possess underlying causal relationships. Notably, no common metabolites emerged from the reverse MR analysis. Moreover, after FDR correction, metabolic pathway analysis identified 40 significant pathways across 11 stroke phenotypes.

**Conclusions:**

The identified metabolites and their associated metabolic pathways are promising circulating metabolic biomarkers, holding potential for their application in stroke screening and preventive strategies within clinical settings.

**Supplementary Information:**

The online version contains supplementary material available at 10.1186/s12967-023-04677-4.

## Introduction

Stroke is one of the most prevalent neurological disorders and is a major cause of disability and death among middle-aged and elderly individuals, posing a significant public health concern on a global scale [1]. According to the Global Burden of Disease estimation in 2019, stroke incidence was 12.2 million cases, the prevalent cases of stroke were 101 million, the number of disability-adjusted life-years was 143 million, and the number of deaths caused by stroke was 6.55 million[2]. Stroke has various subtypes, with ischemic stroke most commonly involved. Ischemic stroke can be further divided into three subtypes: large artery stroke (LAS), cardioembolic stroke (CES), and small vessel stroke (SVS) [3]. Furthermore, stroke includes intracerebral hemorrhage (ICH) and subarachnoid hemorrhage (SAH) [4]. Transient ischemic attack (TIA) is a robust predictor of stroke and is considered a minor stroke [5]. White matter hyperintensities (WMH) and brain microbleeds (BMB) are important risk factors for ischemic stroke [6] and ICH [7]. While the pathological processes vary among different stroke subtypes, they all involve the death of nerve cells [8]. Despite several studies on the nature of stroke, the biological mechanisms and risk factors underlying its occurrence remain unclear. Identifying modifiable risk factors for stroke is crucial for developing preventative interventions.

Recently, the connection between metabolomics and stroke has gained attention. Metabolomics is used for biomarker discovery, providing insights into the processes of disease occurrence and progression by uncovering altered metabolic pathways and intermediate metabolites [9]. Metabolites are the end products or intermediate compounds in metabolism that provide essential functions in the human body. Multiple studies have demonstrated that metabolites are functional intermediates that can elucidate the potential biological mechanisms underlying disease genetics [10, 11]. Metabolite alteration may play important roles as both etiological factors and therapeutic targets for various conditions [12].

With the ongoing advancements in genetics, genome-wide association studies (GWASs) have played a crucial role in stroke research [13]. The GWAS is a method that involves scanning the entire genome of individuals to identify common genetic variants associated with specific traits or diseases. Currently, GWAS research has successfully identified 32 genetic loci associated with an increased risk of stroke and its various subtypes [14]. Some genetic variants identified through GWAS are associated with different aspects of stroke risk, including blood pressure, venous thromboembolism, and lipid metabolism, all of which are relevant to the pathophysiology of stroke [14, 15]. Additionally, GWAS has revealed new loci unrelated to stroke pathophysiology, which may be involved in other biological processes. Furthermore, metabolomics advancements have made it possible to measure hundreds of circulating metabolites and conduct GWASs in large population cohorts [16–18]. However, translating these genetic findings into the underlying biological mechanisms of stroke occurrence and development encounters significant challenges. To enhance our understanding of the biological mechanisms of stroke, further in-depth analysis is required to unravel the causal interactions between serum metabolites and susceptibility to stroke.

Due to limitations in sample size, residual confounding, and the potential for reverse causality in observational studies, the causal relationship between blood metabolites and stroke cannot be determined conclusively. While clinical randomized trials [19] provide the most robust method to evaluate study findings, assessing the correlation between serum metabolites and stroke poses challenges due to cost constraints and ethical considerations in participant recruitment.

Mendelian randomization (MR) has emerged as a popular alternative method recently for assessing the causal effects of factors on diseases while minimizing biases arising from confounding factors or reverse causality [20]. MR analysis utilizes individual genetic variation, randomly distributed during conception, as an instrumental variable [21]. Through leveraging instrumental variable data from extensive GWAS and identified single nucleotide polymorphisms (SNPs) associated with serum metabolites, MR analysis establishes the causal correlation between exposures and outcomes.

Previous studies have utilized MR analysis to assess the stroke risk with metabolites [22–24]. Nevertheless, they have been limited in their comprehensiveness. Contrary to the relatively small number of metabolites studied in previous investigations, we increased the scope to 486. Furthermore, the outcomes considered in previous studies were more limited than ours, including 11 different outcomes. Moreover, our study has a larger sample size than similar studies. Furthermore, while we focused mostly on Europeans, earlier research included various population types as exposure sources, which may have biased the results. Although these studies all employed MR methodology, our study offers a more comprehensive and in-depth analysis. The objective of this article is to implement a two-sample MR approach to: [1] assess the causal effects of human serum metabolites on stroke, [2] identify common metabolites with causal effects on multiple stroke subtypes, and [3] identify potential metabolic pathways that may contribute to understanding the mechanisms underlying stroke occurrence. Our study findings can lay the groundwork for future research directions in stroke.

## Materials and methods

### Study design

**Fig. 1:**
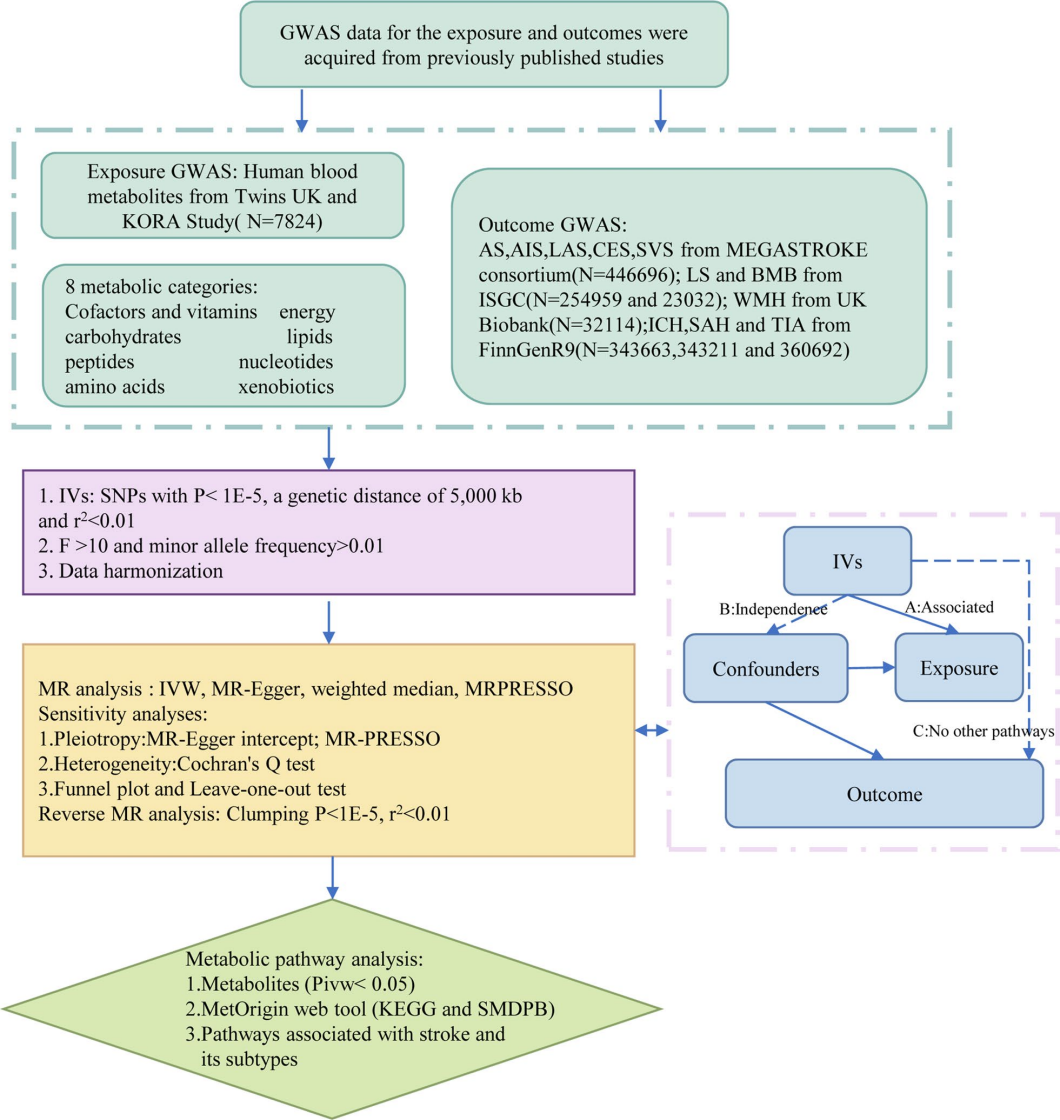
A schematic summary of the study. *GWAS* genome-wide association study, *AS* any stroke, *AIS* any ischemic stroke, *LAS* large artery stroke, *CES* cardioembolic stroke, SVS small vessel stroke, *LS* lacunar stroke, *ICH* intracerebral hemorrhage, *SAH* subarachnoid hemorrhage, *TIA* transient ischemic attack, *WMH* white matter hyperintensities, *BMB* brain microbleeds, *IVs* instrumental variable, *SNPs* single-nucleotide polymorphisms, *MR* Mendelian randomization, *IVW* inverse-variance-weighed,*MR-PRESSO* MR pleitropy residual sum and outlier

The MR study was applied to examine the causal relationship between serum metabolites and stroke. Figure 1 provides a schematic summary of the study design. This study adheres to the reporting guidelines outlined in STROBE-MR (Additional File 1) [25]. The MR design should meet three necessary conditions (Fig. 1): (A) The genetic variants chosen as instrumental variables (IVs) should be strongly correlated with serum metabolites; (B) The genetic instruments should be unrelated to the outcome of stroke and independent of potential confounding factors; (C) The genetic variant should be specifically associated with stroke through serum metabolites rather than other pathways.

### Data sources on the serum metabolites

The comprehensive summary statistics of genetic influence on human serum metabolites in the Twins UK and KORA studies provide extensive data for GWAS on the human metabolome. The dataset includes genome-wide genotyping data from 7824 European participants, and 486 metabolite concentrations were tested in the GWAS [26]. Among the 486 metabolites, 309 are known, while 177 are unknown. According to the Kyoto encyclopedia of genes and genomes (KEGG) database, the known metabolites can be assigned to eight broad metabolic categories: cofactors and vitamins, energy, lipids, nucleotides, peptides, amino acids, carbohydrates, and xenobiotics. Herein, we excluded 34 metabolite traits that could not be assigned IVs, leaving us with a subset of 452 serum metabolites for further analysis.

### Instrumental variables selection

Herein, we selected SNPs with p-values below the locus-wide significance level (1 × 10^–5^) in the initial analysis as IVs to obtain comprehensive results and enhance sensitivity to IVs. Subsequently, all IVs underwent linkage disequilibrium (LD) clumping (r^2^ = 0.01; distance = 5000 kb) to mitigate the influence of correlated SNPs. Furthermore, Phenoscanner (http://www.phenoscanner.medschl.cam.ac.uk/) was screened to identify the potential pleiotropic effects. Additionally, we calculated the F-statistic [R^2^ (N–2)/(1–R^2^)], which assesses the strength of each instrument, where R^2^ represents the proportion of variance explained by the genetic instrument, and N is the effective sample size of GWAS [27]. The SNPs with an F-statistic threshold greater than ten were chosen for the subsequent MR analysis as they provided a reliable estimate of genetic variation [28]. Finally, we excluded palindromic SNPs [29] (where the effective allele is unclear) from our study.

### Data sources on the stroke and its subtypes

Stroke is classified based on the clinical criteria defined by the World Health Organization (WHO) and the tenth edition of the International Classification of Diseases (ICD-10) [30]. Data for certain stroke subtypes were sourced from publicly available summary data provided by the MEGASTROKE consortium [14]. The MEGASTROKE consortium encompassed 446,696 individuals of European ancestry (40,585 any stroke (AS) cases and 406,111 controls). Within any ischemic stroke (AIS) category, there were 34,217 cases of overall AIS, 4,373 cases of LAS, 7,193 cases of CES, and 5,386 cases of SVS. Although the MEGASTROKE study included results for SVS, the cases were defined based on Trial of Org 10172 in Acute Stroke Treatment criteria [3] and did not specifically focus on MRI findings. Therefore, we conducted a study focusing on small vessel infarction using a sample of recent lacunar stroke (LS) cases, comprising 6,030 cases and 248,929 controls [31]. The WMH and BMB were imaging markers of cerebral microstructural damage [32]. The WMH is an increased brightness on T2-weighted brain images [33]. The BMBs are small, low-signal lesions identified on magnetic susceptibility-weighted imaging sequences or T2-weighted gradient-recalled echo sequences [34]. The summary data for WMH (N = 32,114) were derived from an expanded set of a recent GWAS study of brain imaging phenotypes conducted by the UK Biobank [35]. For BMBs, we could not find a GWAS specifically focused on individuals of European ancestry. However, in a recent multi-ethnic GWAS study on BMBs [36], we identified 2889 cases of microbleeds among the remaining 23,032 individuals after excluding patients with dementia and stroke. Summary-level data for the remaining stroke subtypes were generated from the latest FinnGen R9 Biobank [37], which included 3,749 cases of ICH, 3289 cases of SAH, and 18,398 cases of TIA. Further information on the GWAS can accessed at (https://www.finngen.fi/en). The studies in these consortia obtained approval from local research ethics committees and institutional review boards, and all participants provided written informed consent. Table 1 shows the characteristics of summarized datasets for the stroke subtypes.

**Table 1:** Characteristics of the summary datasets for stroke

Stroke subtypes	Ethnicity	Sample size	Cases	Control
Any stroke	European	446,696	40,585	406,111
Any ischemic stroke	European	446,696	34,217	412,479
Large artery stroke	European	446,696	4373	442,323
Cardioembolic stroke	European	446,696	7193	439,503
Small vessel stroke	European	446,696	5386	441,310
Lacunar stroke	European	254,959	6030	248,929
White matter hyperintensities	European	32,114	–	–
Brain microbleed	Mixed	23,032	2889	20,143
Intracerebral hemorrhage	European	343,663	3749	339,914
Subarachnoid hemorrhage	European	343,211	3289	339,922
Transient ischemic attack	European	360,692	18,398	342,294

### MR analysis

Herein, a two-sample MR analysis was utilized to evaluate the causal correlation between serum metabolites and stroke and its subtypes. Subsequently, the fixed-effects or random-effects IVW method was employed as the primary MR analysis. The choice between the fixed-effects and random-effects IVW methods depends on heterogeneity and pleiotropy. The fixed-effects IVW model estimates are given higher significance when neither heterogeneity nor pleiotropy exists. In cases of heterogeneity without pleiotropy, we favor the random-effects IVW model. The random-effects IVW method is chosen for its ability to provide unbiased estimates by accounting for potential horizontal pleiotropy and striving to achieve balance in this context [38]. To enhance the robustness of results, we employed the MR-Egger method, weighted median analysis, and MR pleiotropy residual sum and outlier (MR-PRESSO) test as sensitivity analysis methods. The MR-Egger method considers directional horizontal pleiotropic effects. Whenever the intercept term significantly deviates from zero, it indicates the presence of invalid instruments and suggests potential bias in the IVW method [39]. The I^2^ value and Q-test assessed the potential heterogeneity and identified outliers in the IVW and MR-Egger analyses. The weighted median analysis requires at least half of the instruments to be valid, and the final overall MR estimate is determined by taking the median of causal estimates from each SNP [40]. The MR-PRESSO test was also conducted to identify potential horizontal pleiotropy and correct for its impact by removing outliers [41]. Additionally, we performed leave-one-out analyses to further evaluate the robustness of associations observed by individual SNP drivers.

Herein, a p-value less than 0.05 was considered a nominal association. False Discovery Rate (FDR) correction was employed to control for false positives in multiple tests [42]. Associations were considered statistically significant if the estimated causal effect of a given metabolite had an FDR value of < 0.05. The statistical power (> 80%) was estimated using the mRnd power calculator (http://cnsgenomics.com/shiny/mRnd/) [43]. The statistical analyses were performed using R software version 4.2.3. The MR analyses were performed using the TwoSampleMR package and the MRPRESSO package.

### Metabolic pathway analysis

Metabolic pathways were analyzed via the web-based MetOrigin**.** (http://metorigin.met-bioinformatics.cn/) [44]. Herein, we utilized two databases, the Small Molecule Pathway Database (SMPDB) and the KEGG. Subsequently, functional enrichment and pathway analyses were conducted to identify metabolite groups or pathways potentially associated with the biological processes underlying stroke. The significance level for the pathway analysis was set at 0.05. Furthermore, this study only analyzed the metabolites that passed the recommended association threshold (P < 0.05) determined by IVW.

## Results

### Selection of IVs

The analysis encompassed a spectrum of 3 to 222 chosen IVs for serum metabolites. (Additional file 3: Table S1). These IVs contributed to a minimum of 0.239% variance in their corresponding metabotypes. Importantly, the lowest F-statistic value found in validity testing was 17.64, exceeding the threshold of 10, indicating a negligible probability of encountering weak instrument bias. Additional file 7: Table S5 shows the detailed information on all SNPs**. **Additional file 6: Table S4 presents the genetic associations between significant metabolite SNPs and their respective outcomes (PhenoScanner).

### Causal effects of serum metabolites on 11 stroke phenotypes

**Fig. 2:**
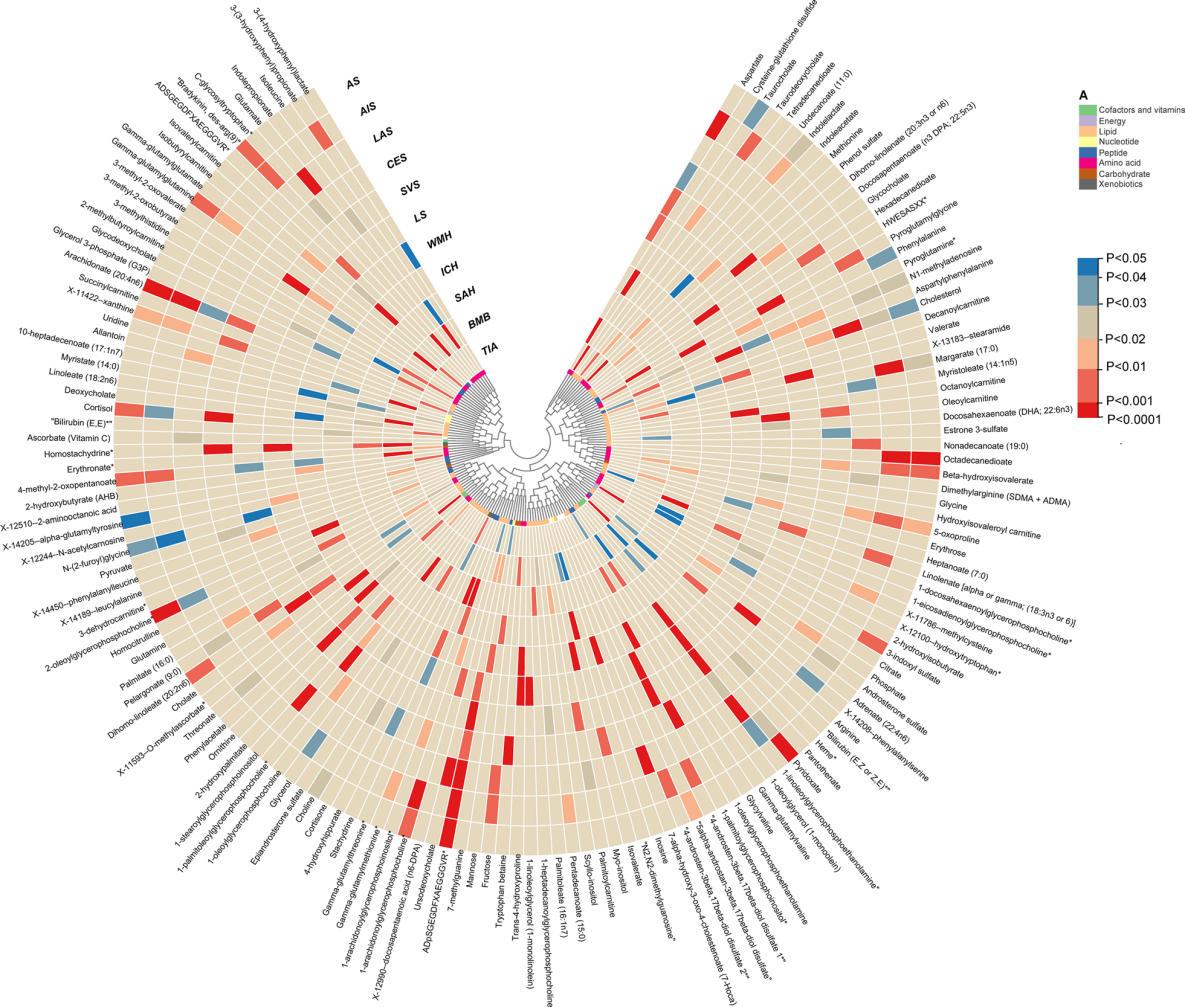
Mendelian randomization asssociations between serum metabolites and 11 stroke phenotypes

**Fig. 3:**
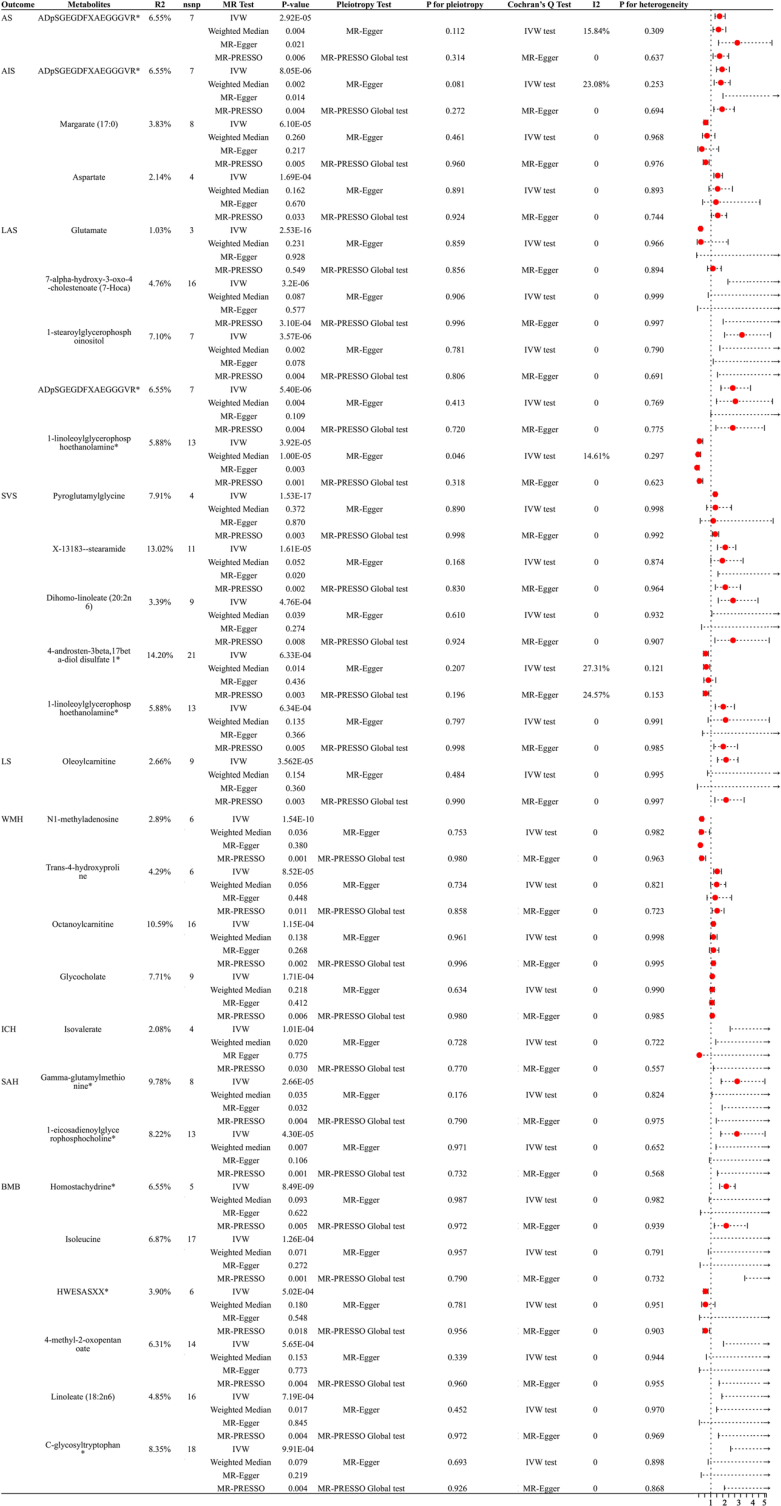
Sensitivity analysis of significant casual associations in stroke phenotypes

The unknown metabolites were excluded from the analysis for a better presentation of the results. The IVW method revealed 266 causative associations (p < 0.05) between serum metabolites and 11 stroke phenotype traits (Additional file 4: Table S2). These associations correspond to 151 specific metabolites **(**Fig. 2**)**. After FDR correction, we observed 25 metabolites with significant causative correlations to stroke and its subtypes (FDR < 0.05), including 13 metabolites from the lipids pathways, 1 from nucleotides pathways, 4 from the peptides pathways, 6 from the amino acids pathways and 1 from the xenobiotic pathways (Fig. 3). The results were as follows:

AS: The IVW results indicated that genetically predicted higher concentrations of ADpSGEGDFXAEGGGVR* (OR: 1.599, 95% CI 1.283–1.993, p = 2.92 × 10^–5^, FDR = 0.0044) were associated with higher AS risk.

AIS: The IVW results demonstrated that genetically predicted higher concentrations of ADpSGEGDFXAEGGGVR* (OR: 1.776, 95% CI 1.380–2.285, p = 8.05 × 10^–6^, FDR = 0.0036), Aspartate (OR: 1.471, 95% CI 1.203–1.799, p = 1.70 × 10^–4^, FDR = 0.0255) were associated with a higher risk of AIS. Conversely, Margarate (17:0) (OR: 0.628, 95% CI 0.499–0.788, p = 6.10 × 10^–5^, FDR = 0.0138) was correlated with a reduced risk of AIS.

LAS: 7-alpha-hydroxy-3-oxo-4-cholestenoate (7-Hoca) (OR: 3.852, 95% CI 2.184–6.794, p = 3.20 × 10^–6^, FDR = 0.0007), 1-stearoylglycerophosphoinositol (OR: 3.166, 95% CI 1.945–5.154, p = 3.57 × 10^–6^, FDR = 0.0005) and ADpSGEGDFXAEGGGVR* (OR: 2.522, 95% CI 1.693–3.157, p = 5.40 × 10^–6^, FDR = 0.0006) were associated with a higher risk of LAS. Glutamate (OR: 0.271, 95% CI 0.199–0.371, p = 2.53 × 10^–16^, FDR < 0.0001) and 1-linoleoylglycerophosphoethanolamine* (OR: 0.198, 95% CI 0.091–0.428, p = 3.92 × 10^–5^, FDR = 0.003) were correlated with a reduced risk of LAS.

CES, TIA: After multiple-testing correction, we found a nonsignificant causal relationship between metabolites and CES and TIA.

SVS: Pyroglutamylglycine (OR: 1.297, 95% CI 1.222–1.377, p = 1.53 × 10^–17^, FDR < 0.0001), X-13183—stearamide (OR: 1.997, 95% CI 1.458–2.734, p = 1.61 × 10^–5^, FDR = 0.0036), dihomo-linoleate (20:2n6) (OR: 2.539, 95% CI 1.505–4.281, p = 4.76 × 10^–4^, FDR = 0.0410) and 1-linoleoylglycerophosphoethanolamine*(OR: 1.855, 95% CI 1.301–2.643, p = 6.34 × 10^–4^, FDR = 0.0410) were associated with a higher risk of SVS. 4-androsten-3beta,17beta-diol disulfate 1*(OR: 0.624, 95% CI 0.476–0.818, p = 6.33 × 10^–4^, FDR = 0.0410) was correlated with a reduced risk of SVS.

LS: Oleoylcarnitine (OR: 2.052, 95% CI 1.460–2.886, p = 3.56 × 10^–5^, FDR = 0.0160) was associated with an increased risk of LS.

WMH: N1-methyladenosine (OR: 0.205, 95% CI 0.126–0.333, p = 1.53 × 10^–10^, FDR < 0.0001) was associated with a lower risk of WMH. Trans-4-hydroxyproline (OR: 1.530, 95% CI 1.237–1.891, p = 8.52 × 10^–5^, FDR = 0.0192), Octanoylcarnitine(OR: 1.204, 95% CI 1.096–1.324, p = 1.15 × 10^–4^, FDR = 0.0193) and Glycocholate (OR: 1.114, 95% CI 1.053–1.179, p = 1.71 × 10^–4^, FDR = 0.0193) were associated with an increased risk of WMH.

ICH: Isovalerate (OR: 7.130, 95% CI 2.648–19.199, p = 1.01 × 10^–4^, FDR = 0.0229) was associated with an elevated risk of ICH.

SAH: Gamma-glutamylmethionine* (OR: 3.251, 95% CI 1.876–5.635, p = 2.66 × 10^–5^, FDR = 0.0060) and 1-eicosadienoylglycerophosphocholine*(OR: 3.224, 95% CI 1.840–5.650, p = 4.30 × 10^–5^, FDR = 0.0064) were an increased risk of SAH.

BMB: Homostachydrine* (OR: 2.312, 95% CI 1.738–3.076, p = 8.49 × 10^–9^, FDR < 0.0001), Isoleucine (OR: 34.649, 95% CI 5.659–212.142, p = 1.26 × 10^–4^, FDR = 0.0283).

4-methyl-2-oxopentanoate (OR: 5.484, 95% CI 2.084–14.430, p = 5.65 × 10^–4^, FDR = 0.0484), Linoleate (18:2n6) (OR: 4.834, 95% CI 1.940–12.045, p = 7.19 × 10^–4^, FDR = 0.0484) and C-glycosyltryptophan* (OR:11.483, 95% CI 2.686–49.088, p = 9.91 × 10^–4^, FDR = 0.0497) were an elevated risk of BMB. HWESASXX* (OR: 0.523, 95% CI 0.363–0.754, p = 5.02 × 10^–4^, FDR = 0.0484) was correlated with a lower risk of BMB.

Moreover, it was observed that certain stroke phenotypes shared common causative metabolites among the remaining associations. After FDR correction, the p-values presented for the following results are greater than 0.05. However, they are based on the original values. The ADpSGEGDFXAEGGGVR* indicated a strong causal relationship withAS, AIS and LAS, as well as a suggestive causal association with CES (OR = 2.096, 95% CI 1.162–3.780, p = 0.01395), SVS (OR = 2.254, 95% CI 1.348–3.770, p = 0.00196), LS (OR = 1.984, 95% CI 1.177–3.343, p = 0.0101) and BMB (OR = 1.750, 95% CI 1.092–2.807, p = 0.0202). The 1-linoleoylglycerophosphoethanolamine* demonstrated a robust causal relationship with LAS and SVS and also exhibited suggestive associations with AIS (OR = 0.676, 95% CI 0.464–0.984, p = 0.0410), CES (OR = 0.505, 95% CI 0.273–0.935, p = 0.0298), LS (OR = 2.062, 95% CI 1.340–3.172, p = 9.89 × 10^–4^), and WMH (OR = 1.614, 95% CI 1.220–2.137, p = 8.17 × 10^–4^). However, the correlation observed in AIS and CES differs from the rest of the associations. The N1-methyladenosine exhibited a robust causal correlation with WMH, while showing an inverse relationship with AS (OR: 3.056, 95% CI 1.145–8.156, p = 0.0258), AIS (OR: 2.989, 95% CI 1.139–7.838, p = 0.0261), CES (OR: 30.198, 95% CI 1.893–481.823, p = 0.0159), SVS (OR: 3.804, 95% CI 1.268–11.412, p = 0.0171), and LS (OR: 10.295, 95% CI 1.086–97.607, p = 0.0422). Aspartate exhibited causal associations with AIS, CES (OR: 2.031, 95% CI 1.041–3.961, p = 0.0377), SVS (OR: 2.577, 95% CI 1.228–5.405, p = 0.0123), LS (OR: 2.981, 95% CI 1.273–6.976, p = 0.0118), and BMB (OR: 0.363, 95% CI 0.182–0.724, p = 0.004). Homostachydrine* exhibited the same causal associations with BMB and SAH (OR: 1.686, 95% CI 1.137–2.501, p = 0.0094) while demonstrating an inverse causal relationship with CES (OR: 0.689, 95% CI 0.535–0.887, p = 0.0038) and LS (OR: 0.533, 95% CI 0.362–0.785, p = 0.0015). Dihomo-linoleate (20:2n6) displayed causal associations with both SVS and AS (OR = 1.276,95% CI 1.049–1.551, p = 0.0146), as well as LS (OR = 2.033, 95% CI 1.171–3.528, p = 0.0117). Isovalerate demonstrated causal associations with ICH, CES (OR = 0.277, 95% CI 0.109–0.704, p = 6.997 × 10^–3^), and BMB (OR = 3.134, 95% CI 1.033–9.512, p = 0.0437). Margarate (17:0) exhibited causal associations with both AIS, AS (OR: 0.735, 95% CI 0.558–0.967, p = 0.0281), and BMB (OR: 3.328, 95% CI 1.423–7.778, p = 5.52 × 10^–3^). Oleoylcarnitine displayed causal associations with LS and CES (OR: 1.763, 95% CI 1.144–2.717, p = 0.0101). The 1-stearoylglycerophosphoinositol had causal associations with LAS and WMH (OR: 1.129, 95% CI 1.026–1.242, p = 0.0126). The 4-androsten-3beta,17beta-diol disulfate 1* demonstrated causal associations with both SVS, AIS (OR: 0.804, 95% CI 0.676–0.957, p = 0.0142) and LS (OR: 0.643, 95% CI 0.490–0.843, p = 0.0014). Gamma-glutamylmethionine* showed causal associations with both SAH and AIS (OR: 0.724, 95% CI 0.547–0.958, p = 0.0237). Pyroglutamylglycine exhibited causal associations with SVS, AIS (OR: 1.281, 95% CI 1.071–1.534, p = 0.0069), ICH (OR: 2.129, 95% CI 1.313–3.453, p = 0.0022) and TIA (OR: 1.214, 95% CI 1.039–1.419, p = 0.0148). Isoleucine had causal associations with BMB and SAH (OR: 0.398, 95% CI 0.159–0.997, p = 0.0492). Trans-4-hydroxyproline indicated causal associations with WMH and LS (OR: 2.195, 95% CI 1.376–3.500, p = 9.70 × 10^–4^). This study revealed that certain metabolites were observed across multiple outcomes. However, none demonstrated statistical significance after multiple corrections. For example, cholesterol exhibited significance in AS, AIS, LAS, CES, and TIA; however, these findings lost statistical significance following multiple corrections. Other metabolites followed the same patterns but were not elaborated on individually.

### Sensitive analysis

The IVW method is susceptible to weak instrument bias, prompting us to conduct a series of sensitivity analyses. Figure 3 illustrates the results of all sensitivity analyses demonstrating significant causal relationships between 25 metabolites and stroke phenotypes. After completing sensitivity analyses, we found that just three of the 25 metabolites had statistically significant relationships with their respective outcomes (P < 0.05). Specifically, these three metabolites were ADpSGEGDFXAEGGGVR* for AS and AIS, 1-linoleoylglycerophosphoethanolamine* for LAS, and Gamma-glutamylmethionine* for SAH. Generally, causal associations are considered robust when statistical significance (p < 0.05) is observed in both the weighted median test and the MR-PRESSO test [45]. Therefore, an additional 7 metabolites were deemed robust, including were 1-stearoylglycerophosphoinositol and ADpSGEGDFXAEGGGVR* for LAS, Dihomo-linoleate (20:2n6) and 4-androsten-3beta,17beta-diol disulfate 1* for SVS, N1-methyladenosine for WMH, Isovalerate for ICH, 1-eicosadienoylglycerophosphocholine* for SAH, and Linoleate (18:2n6) for BMB. Furthermore, certain metabolites exhibited significance in the IVW method and MR-PRESSO test, even though they did not show significance in the weighted median test, indicating potential causal associations. Consequently, only glutamate for LAS did not meet our established criteria among the remaining significant metabolites. In all metabolic outcomes, MR-Egger regression did not uncover evidence of horizontal pleiotropy, and Cochran's Q test did not reveal evidence of heterogeneity. No significant horizontal directional pleiotropy was detected by MR-PRESSO (Additional file 4: Table S2 and Additional file 6: S4). The leave-one-out analysis was shown in Additional file 2.

### Reverse MR analysis

**Fig. 4:**
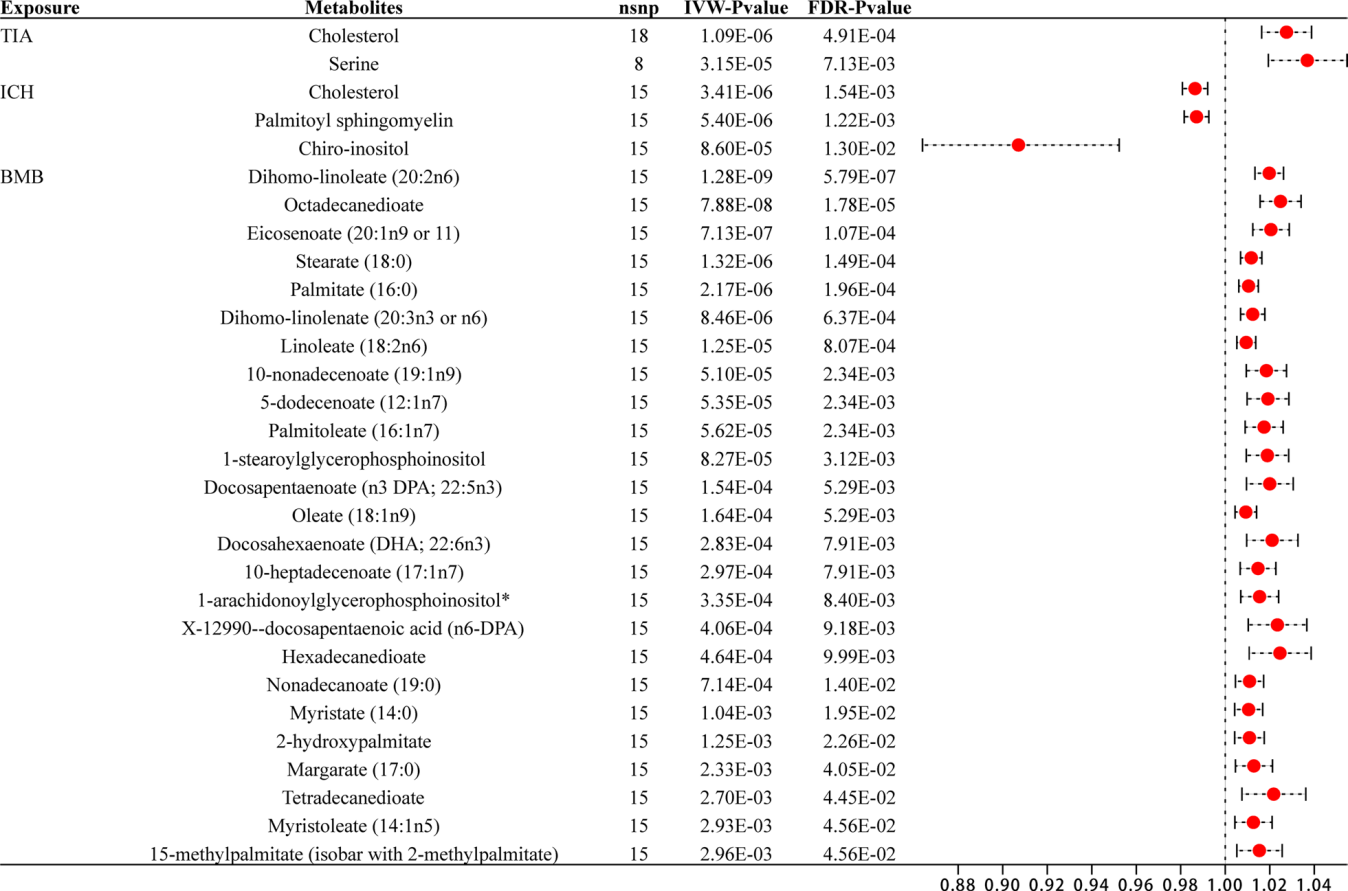
The significant casual relationships between stroke phenotypes and metobolites

Additionally, a reverse MR analysis was conducted to explore the potential causal effects of stroke phenotypes on serum metabolites. This analysis followed the previously described methodology. Our study results displayed that after multiple corrections, 30 significant associations were found between metabolites and stroke phenotypes. These 30 associations involve 29 different metabolites, with corresponding outcomes being TIA, ICH, and BMB (Additional file 5: Table S3). Compared to our positive MR results, these divergent discoveries suggest the absence of reverse causality. Figure 4 depicts the significant causal relationships between stroke phenotypes and metabolites.

### Metabolic pathway analysis

**Fig. 5:**
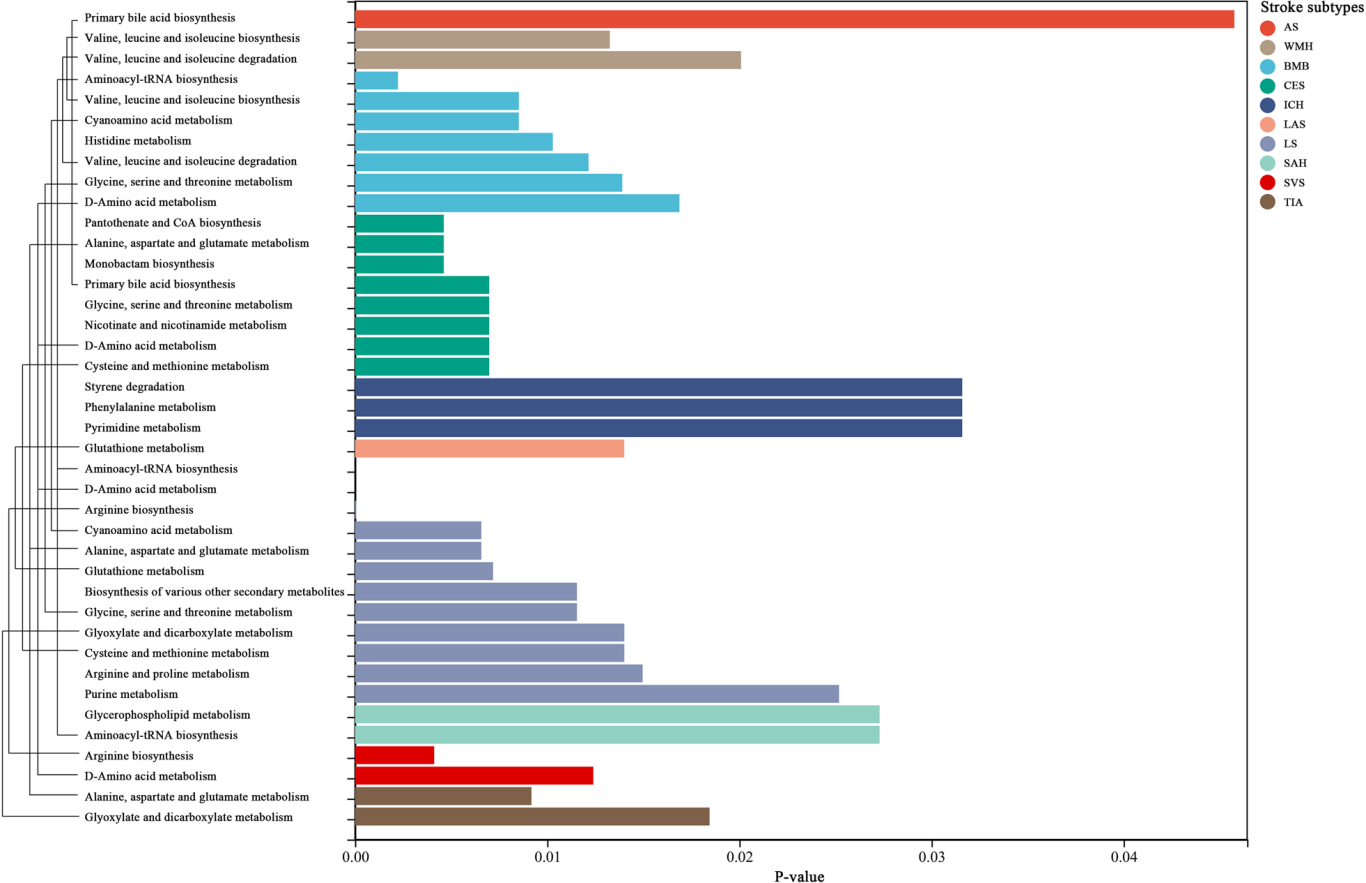
The 40 significant metabolic pathways associated with stroke subtypes

A total of 118 metabolic pathways were integrated with the metabolites related to stroke phenotypes, and 40 significant metabolic pathways remained after FDR correction (Fig. 5**)**. There are five metabolic pathways associated with AS, with the most significant pathway being “primary bile acid biosynthesis” (p = 2.86 × 10^–3^, FDR = 4.58 × 10^–2^). Among the seven metabolic pathways related to AIS, the most notable is “steroid degradation” (p = 3.25 × 10^–2^, FDR = 0.114). For LAS, the most significant pathway among the nine identified is “glutathione metabolism” (p = 6.67 × 10^–4^, FDR = 0.0140). In the case of CES, among the 31 metabolic pathways, the most significant one is “pantothenate and CoA biosynthesis” (p = 2.85 × 10^–4^, FDR = 4.62 × 10^–3^). Similarly, for SVS, “arginine biosynthesis” is the most significant of the 15 pathways investigated for SVS (p = 2.06 × 10^–4^, FDR = 4.12 × 10^–3^). Among the 17 metabolic pathways correlated with LS, the most significant is “aminoacyl-tRNA biosynthesis” (p = 7.85 × 10^–9^, FDR = 1.78 × 10^–7^). Among the four identified pathways for WMH, the most prominent one is “valine, leucine and isoleucine biosynthesis”(p = 9.47 × 10^–4^, FDR = 0.0133). Conversely, for ICH, the most significant pathway within the three revealed pathways is "styrene degradation" (p = 1.15 × 10^–2^, FDR = 0.0316). The most significant metabolic pathway among the seven SAH-related pathways is “glycerophospholipid metabolism” (p = 3.37 × 10^–3^, FDR = 0.0273). Moreover, "aminoacyl-tRNA biosynthesis" (p = 7.98 × 10^–5^, FDR = 2.23 × 10^–3^) has the highest level of significance when compared to the other 11 pathways related to BMB. Finally, among the nine pathways related to TIA, "alanine, aspartate and glutamate metabolism" (p = 5.10 × 10^–4^, FDR = 9.18 × 10^–3^) emerges as the most statistically significant. Additionally, it was observed that certain stroke phenotypes may share common metabolic pathways. For instance, the “steroid degradation” pathway for AS, AIS, LAS, CES, and TIA. The “pantothenate and CoA biosynthesis” pathway for AIS, CES, SVS, LS, SAH, and BMB. The “valine, leucine, and isoleucine biosynthesis” for AS, AIS, CES, SVS, WMH, SAH, and BMB. The “arginine biosynthesis” pathway for AIS, LAS, CES, SVS, LS, SAH, BMB, and TIA. Additionally, there are some shared pathways that we will not elaborate on in detail, and Additional file 6: Table S4 describes all details**.**

## Discussion

**Fig. 6:**
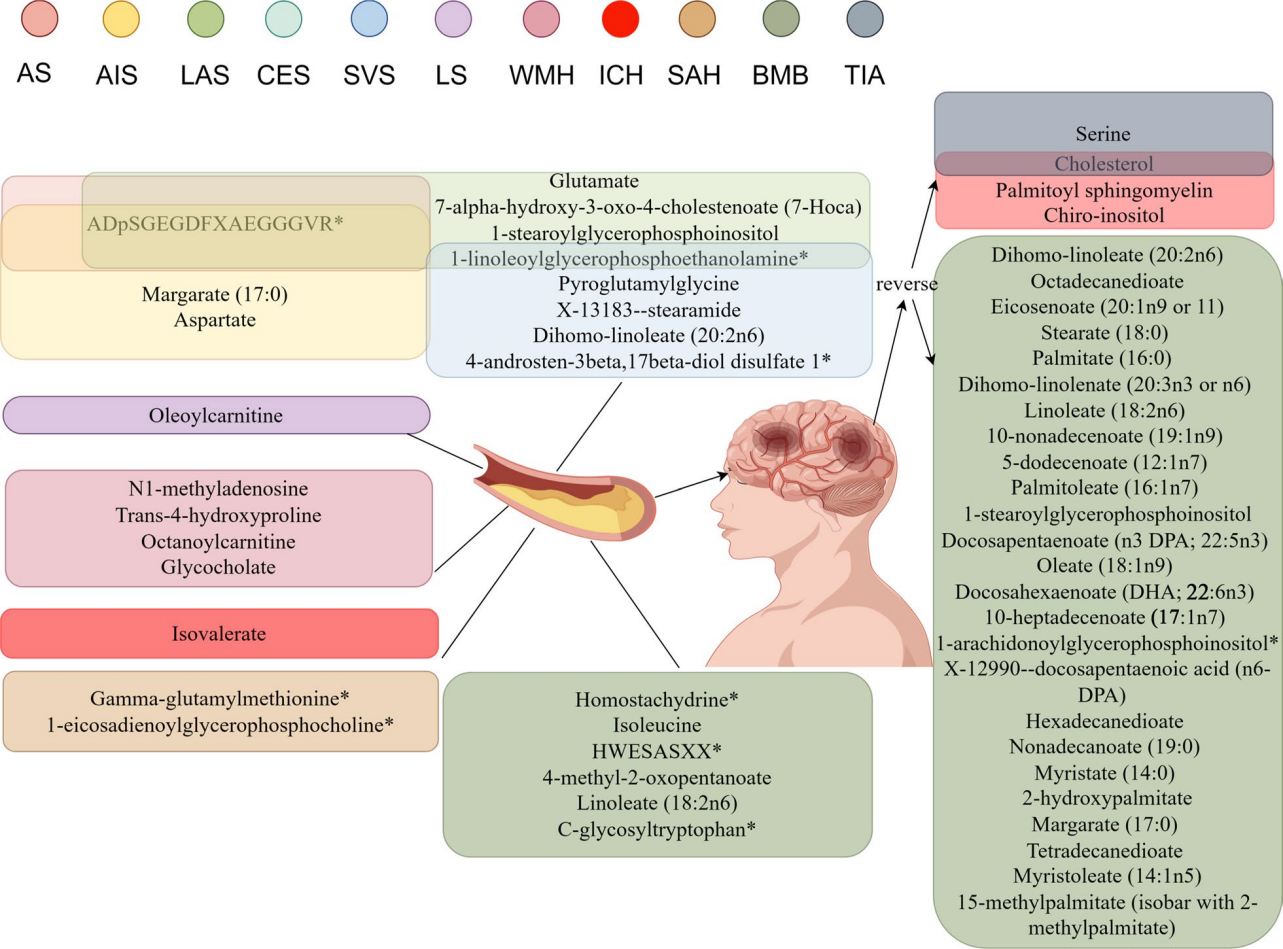
Bidirectional casual links between metabolits an stroke subtypes

Stroke pathogenic pathways are now better understood owing to the rapid advancements in metabolome studies over the past few decades. Most studies are animal or case–control studies, which can demonstrate an association with stroke but cannot establish a causal relationship. In our MR study, we excluded unknown metabolites and established 28 associations involving 25 metabolites, which were observed with multiple-testing corrected significance. In the reverse analysis, we found 30 associations involving 29 metabolites, which also passed multiple tests (Fig. 6). Furthermore, our analysis identified 40 significant metabolic pathways intricately linked to the 11 investigated stroke phenotypes. These findings expand our knowledge and pave the way for future advances in precision therapeutics and early diagnostic techniques by unraveling the intricate interplay of gene-environment interactions in stroke pathogenesis.

There is a consensus that metabolomics will play a pivotal role in shaping future stroke therapeutic strategies due to the continuous expansion of novel metabolic insights and advancements in metabolomics research, as well as the ongoing discovery of metabolites and metabolic pathways associated with stroke [11, 46]. Blood is the most used sample source for metabolomics identification because it contains numerous detectable metabolites and can be easily obtained in large sample sizes, facilitating the screening of circulating biomarkers for stroke risk [47]. Current metabolomics studies have revealed alterations in the metabolic profiles of stroke patients, with the most reported metabolites being amino acids, lipids, polyamines, and nucleotides [48]. Our study has confirmed the existence of stroke-specific metabolic profiles and identified crucial metabolites and metabolic pathways correlated with the onset of stroke and its related phenotypes.

Herein, four metabolites from the peptide metabolism pathways were identified to have been causally associated with stroke pathogenesis. Peptides are vital in human physiology, serving as hormones, neurotransmitters, growth factors, and ion channel ligands. Generally, peptides were selective and efficient signaling molecules bound to specific cell surface receptors, including ion channels or G-protein coupled receptors (GPCRs, where they initiated intracellular effects [49]. The role of peptides in diseases is also receiving increasing attention. Our findings revealed that ADpSGEGDFXAEGGGVR* was a fibrinogen cleavage peptide in multiple stroke phenotypes, indicating its potential pathogenicity. It had previously been correlated with prostate cancer and osteoarthritis [45, 50]. A nationwide study reported that gamma-glutamyl transferase is a novel biomarker predicting stroke risk [51]. Here, we observed a significant presence of gamma-glutamyl methionine*, and its close correlation with gamma-glutamyl may serve as validation for the aforementioned study. In stroke-related research, endogenous pleiotropic dipeptides like carnosine have been identified as neuroprotective agents [52]. Conversely, our study emphasized the pathogenicity of dipeptide pyroglutamylglycine across various stroke phenotypes. However, the exact mechanism by which these metabolites influenced stroke remains unclear. The development of protective drugs targeting this metabolite could offer valuable insights. Therefore, further research was warranted to delve deeper into this aspect.

Previous studies have demonstrated aberrant lipid metabolism as a significant pathophysiological hallmark in ischemic stroke [53]. According to our results, 13 lipids have been linked to an increased stroke risk. The 1-linoleoylglycerophosphoethanolamine* from the lysolipid pathway was detected across various ischemic stroke outcomes. Notably, phospholipids have been consistently implicated in various complex diseases, including cardiovascular disorders and cancer [54]. Lysophosphatidic acid, a metabolic byproduct of phospholipids, is commonly implicated in various processes and is linked to the formation of atherosclerotic plaques and thrombotic activity [55]. Clinical studies have demonstrated changes in several phospholipid biomarkers, including lysophosphatidylcholine and lysophosphatidylethanolamine, in ischemic stroke patients compared with healthy control subjects [56]. Moreover, it was found that metabolic disorders, including atherosclerosis, diabetes, and neurodegenerative diseases, all share the metabolic pathway of lysophosphatidylcholine metabolism with stroke [57]. This finding partially elucidates the simultaneous occurrence of stroke alongside these conditions. Although the role of linoleoylglycerophosphocholine in stroke has not been previously documented, it is recognized as lysophosphatidylcholine. Previous studies have established its potential as a biomarker associated with insulin resistance and a significant reduction in the risk of type 2 diabetes [58, 59]. Considering the close association between stroke, especially LAS, and SVS, with diabetes [60], investigating the role of 1-linoleoylglycerophosphoethanolamine* potentially leads to promising avenues of research.

The 1-stearoylglycerophosphoinositol and 1-eicosadienoylglycerophosphocholine* from the lysolipid pathway were detected significantly in the LAS and SAH outcomes, respectively. The presence of 1-stearoylglycerophosphoinositol has been found to be associated with leukocyte telomere length, which serves as a predictive marker of biological aging [61]. A study comparing a centenarian group with a younger control group revealed that the depletion of lysolipid stearoyl phosphatidyl choline could be a potential reason for longevity [62]. Herein, 1-stearoylglycerophosphoinositol exhibited a positive correlation with LAS, and a similar association was observed in WMH. This provides a foundation for the aforementioned studies. The 1-eicosadienoylglycerophosphocholine* was only present in the SAH outcome. While no reported findings exist in the existing literature, it holds potential as a predictive indicator.

Isovalerate, X-13183—stearamide, Linoleate (18:2n6) and Margarate (17:0) were categorized as fatty acids. The first three metabolites disclosed significant positive correlations with ICH, SVS, and BMB outcomes, respectively, while the latter exhibited a negative correlation with AIS. Notably, isovalerate was found to be associated with the BMB outcome. A previous study has revealed that elevated plasma levels of isovalerate, a branched short-chain fatty acid, were observed following acute AIS reperfusion therapy, correlating with the severity and disability of stroke [63]. In another case–control study, elevated levels of linoleic acid were noted in patients with hemorrhagic stroke [64]. Our findings seem to provide a potential explanatory link to previous studies. Several studies have suggested the potential benefits of certain long-chain fatty acids in reducing the risk of stroke [65]. Margarate (17:0), a long-chain fatty acid, remains underexplored in the existing literature regarding its associations and effects on stroke. The underlying pathophysiological mechanisms of these four fatty acids remain unclear; hence, further investigations are needed.

Cholesterol belongs to the sterol/steroid pathways. Elevated cholesterol levels induce atherosclerosis development, which is closely associated with ischemic stroke [66]. In this study, we detected cholesterol in multiple outcomes of ischemic stroke. Although it did not maintain statistical significance after multiple corrections, it still holds potential as a latent predictive marker. Furthermore, 7-alpha-hydroxy-3-oxo-4-cholestenoate (7-Hoca) and 4-androsten-3beta,17beta-diol disulfate 1* are from Sterol/Steroid pathways. Although there was limited investigation into them, these compounds may have an impact on atherosclerosis and, consequently, stroke incidence.

Our study observed that within the amino acid pathway, six metabolites were linked to stroke. Previous studies have indicated that decreased levels of branched-chain amino acids (BCAAs) were observed in patients with ischemic stroke. However, whether the reduction in BCAAs represented a causal pathway or an epiphenomenon in ischemic stroke remained to be determined [67]. Isoleucine, along with 4-methyl-2-oxopentanoate, a branched-chain amino acid, was significant in the BMB outcome. While no other significant BCAAs were observed in other outcomes, our pathway analysis revealed an association between the valine, leucine, and isoleucine biosynthesis pathway (BCAA pathways) and various stroke outcomes.

Furthermore, some research indicated that excitatory amino acids, including glutamate and aspartate, contributed to neuronal damage following a stroke [68, 69]. Although our findings aligned with the mentioned research, it is worth noting that glutamate was only significant for the LAS outcome and was unstable after sensitivity analysis. Conversely, aspartate was present across multiple stroke outcomes, potentially signifying a promising therapeutic target. Moreover, the mechanisms underlying the impact of two significant amino acids in our results, C-glycosyltryptophan* and Trans-4-hydroxyproline, on stroke remained incompletely understood, necessitating further research.

The N1-methyladenosine from nucleotide pathways, initially identified in tRNA and rRNA, exhibits its highest abundance in the brain. A previous study compared ischemic and hemorrhagic stroke patients with healthy control subjects and revealed that a significant elevation of N1-methyladenosine was observed in the plasma of stroke patients. This elevation was correlated with the infarction size and the hemorrhage volume, suggesting the potential of N1-methyladenosine as a biomarker for stroke [70, 71]. Herein, N1-methyladenosine demonstrated a role as a risk factor among different ischemic stroke subtypes, confirming the previously mentioned conclusion. Interestingly, it displayed a protective role in the context of WMH. Unfortunately, the absence of recent relevant research in this area makes interpretation more challenging.

Homostachydrine* from xenobiotic pathways is a type of pipecolic acid betaine [72]. Currently, it has been identified that this compound was closely linked to homocysteine [73], which in turn had been associated with ischemic stroke [74]. This linkage indirectly highlights the potential relevance of homostachydrine to ischemic stroke. Herein, homostachydrine manifested as a protective factor in CES and LS, coinciding with findings presented by Guo et al. [23]. However, concerning BMB and SAH, homostachydrine unveiled its role as a risk factor. Currently, pertinent research documenting such associations remains absent, highlighting the need for further research to corroborate these outcomes.

Our MR analysis identified metabolic pathways that exhibit a causal relationship with the development of stroke and its subtypes. Some of these pathways have been extensively documented in related animal models or studies on blood samples. These pathways include primary bile acid biosynthesis, steroid degradation, glutathione metabolism, pantothenate and CoA biosynthesis, arginine biosynthesis, aminoacyl-tRNA biosynthesis, valine, leucine and isoleucine biosynthesis, glycerophospholipid metabolism, alanine, aspartate and glutamate metabolism [67, 75–80], have been identified in studies involving animal models or blood sample. Our study provides a stronger validation of their conclusions and reveals overlapping metabolic pathways among various stroke subtypes. The primary bile acid biosynthesis pathway was significantly associated with AS and CES. This pathway involves metabolites related to cholesterol. A retrospective study has suggested that bile acid excretion disruption may be an independent risk factor for stroke [81]. Considering cholesterol is also present in various stroke outcomes, we can infer that cholesterol may influence the occurrence and progression of stroke by affecting bile acid metabolism. Additionally, the metabolites involved in steroid degradation include cholesterol, and this pathway is associated with multiple stroke outcomes. Therefore, we hypothesize that cholesterol may affect steroid degradation, thereby influencing stroke occurrence. The pathways of glutathione metabolism, pantothenate, and CoA biosynthesis, arginine biosynthesis, aminoacyl-tRNA biosynthesis, and alanine, aspartate, and glutamate metabolism are also present in multiple stroke outcomes involving the metabolites glutamate and aspartate. Aspartate remains stable in our results, allowing us to infer its influence on the mentioned pathways and its impact on stroke progression. However, glutamate is non-robust in our results, indicating the need for further research to validate it.

Additionally, there are differences between the metabolites primarily involved in pathway analysis and those in our significant results. Therefore, there may be other unexplored metabolic pathways. Further research is required to better understand the relationships between metabolites, metabolic pathways, and stroke outcomes. Moreover, we have also discovered the involvement of certain novel pathways in stroke pathogenesis, including “styrene degradation” playing a significant role in ICH and “clavulanic acid biosynthesis” in SAH. The specific mechanisms behind these findings also require further investigation.

The use of drugs can influence changes in the metabolite profile. For example, statin medications lead to extensive lipid alterations and effectively reduce cholesterol levels [82]. Recently, developing neuroprotective peptide drugs has influenced the occurrence of stroke by affecting the metabolism of amino acids in the human body, including glutamate and aspartate [83]. Additionally, some cardiovascular drugs can influence P-glycoprotein, which regulates the absorption and excretion of xenobiotics. The P-glycoprotein is associated with ischemic stroke in mouse models [84]. Therefore, the use of drugs in stroke patients may interfere with measuring metabolites, emphasizing a challenge for specific research on the impact of a particular metabolite on stroke in the future.

Based on our findings and cross-validation with RCT trial results, this provides early predictive factors for future research on the utility of these biomarkers in blood tests for stroke prevention. This study suggests that targeting certain metabolites may be a promising area for future medication development in treating stroke.

Our study has several strengths. First, a major strength of this study lies in its extensive coverage of genetic variables to comprehensively analyze the genetically determined relation between blood metabolites and the eleven stroke phenotypes. Meanwhile, the genome-wide dataset for stroke subtypes genetic variables primarily utilized populations of European ancestry to mitigate potential biases arising from population differences. Second, using bidirectional MR designs largely avoided reverse causation and residual confounding. Third, applying the largest available dataset on various stroke subtypes in the field, along with extensive sensitivity analyses, ensured the robustness of our findings.

Nevertheless, this study has certain limitations that should be acknowledged. First, we leveraged exposure-specific GWAS data and outcomes from publicly available summary data, with potential sample overlaps that might introduce confounding biases. Additionally, the distinct data sources in this study may correspond to different population groups. These samples could exhibit substantial variations in population characteristics, including age, gender, and socioeconomic background. Such distinctions can potentially influence the interpretation of causal estimates and the validity of causal inferences. Second, owing to the relatively limited number of participants in the exposure dataset and the restricted range of metabolite types, some associations between different metabolites and stroke might be missing. Third, the study participants primarily consisted of individuals of European descent, necessitating an assessment of the generalizability of our findings to other populations. Fourth, to enhance the reliability of our findings, we employed multiple correction analyses. However, this approach might overlook potential metabolites causally related to stroke. Fifth, some metabolites and metabolic pathways covered in this study have not been fully elucidated regarding their functions and mechanisms in diseases, which limits our interpretation of the MR analysis results. Lastly, due to the limited variance explained by SNPs or sample size constraints in GWAS results, some of our MR analyses might lack sufficient power to detect small effects. Future investigations utilizing larger GWAS datasets promise to provide enhanced statistical power and more precise assessments of the genetic influences on metabolites. Although MR has assisted in identifying blood metabolites associated with stroke, there remains a need for prospective studies to delve into their potential mechanisms.

## Conclusion

This two-sample MR study revealed the significant role of serum metabolites in the risk of 11 stroke subtypes. Identifying 28 remarkable causal associations between 25 metabolites and 9 stroke phenotypes, 40 significant metabolic pathways in 11 stroke phenotypes, and nominal causal associations of other metabolites contribute to our understanding of the intricate interplay between metabolites and the brain in the development of stroke. Moreover, they offer valuable potential as circulating metabolic biomarkers, holding promise for their application in stroke screening and preventive strategies within clinical settings. These findings contribute to the understanding of biological mechanisms underlying stroke and pave the way for future exploration of targeted therapeutic interventions.

### Supplementary Information

**Additional file 1: **STROBE-MR checklist of recommended items to address in reports of Mendelian randomization studies.

**Additional file 2: **The result for leave-one-out analysis.

**Additional file 3: Table S1**. The Results of all stroke phenotypes.

**Additional file 4: Table S2**. The Results of all stroke phenotypes (P < 0.05).

**Additional file 5: Table S3**. The reverse results of all stroke phenotypes (P < 0.05).

**Additional file 6: Table S4**. The results of significant metabolites, metabolic pathways, power, and metabolite-related features.

**Additional file 7: Table S5**. The relevant SNP results for all stroke phenotypes.

## Data Availability

As the current study used published GWAS summary data, so, the relevant data is publically available. Further inquiries can be directed to the corresponding authors.
