# Supplementary material for: Assessing the causal effect of genetically predicted metabolites and metabolic pathways on stroke
Source: J Transl Med. 2023 Nov 17;21:822. doi: 10.1186/s12967-023-04677-4 (PMC10655369; doi:10.1186/s12967-023-04677-4)
Supplement: Supplementary file 1 — Additional file 1: STROBE-MR checklist of recommended items to address in reports of Mendelian randomization studies. [file 12967_2023_4677_MOESM1_ESM.docx]

**Additional File 1.**

STROBE-MR checklist of recommended items to address in reports of Mendelian randomization studies.

| Item No. | Section | Checklist item | Page | Relevant text from manuscript |
| --- | --- | --- | --- | --- |
| 1 | Title and Abstract | Indicate Mendelian randomization (MR) as the study’s design in the title and/or the abstract if that is a main purpose of the study | 2 | A two-sample Mendelian randomization study was conducted to investigate the causal effects of serum metabolites on stroke and its subtypes. |
|  | Introduction |  |  |  |
| 2 | Background | Explain the scientific background and rationale for the reported study. What is the exposure? Is a potential causal relationship between exposure and outcome plausible? Justify why MR is a helpful method to address the study question | 4-7 | Stroke is a common neurological disorder that disproportionately affects middle-aged and elderly individuals, leading to significant disability and mortality. Human blood metabolites have been discovered to be useful in unraveling the underlying biological mechanisms of neurological disorders. Therefore, we aimed to evaluate the causal relationship between human blood metabolites and susceptibility to stroke. |
| 3 | Objectives | State specific objectives clearly, including pre-specified causal hypotheses (if any). State that MR is a method that, under specific assumptions, intends to estimate causal effects | 2 | We conducted a Mendelian randomization analysis to assess the causal relationship between human blood metabolites and susceptibility to stroke. We examined the risk associated with 11 different stroke phenotypes, including AS, AIS, LAS, CES, SVS, LS, WMH, ICH, SAH, BMB, and TIA. Likewise, we identified 486 serum metabolites as the exposures of interest. |
|  | Methods |  |  |  |
| 4 | Study design and data sources | Present key elements of the study design early in the article. Consider including a table listing sources of data for all phases of the study. For each data source contributing to the analysis, describe the following: |  |  |
|  | a) | Setting: Describe the study design and the underlying population, if possible. Describe the setting, locations, and relevant dates, including periods of recruitment, exposure, follow-up, and data collection, when available. | 7  7-10 | Figure 1 provides a schematic summary of the study design.  Exposure: Table S1  Outcome: We utilized publicly available summary statistics from four independent cohorts of European ancestry: MEGASTROKE consortium, ISGC, UK Biobank and FinnGen (R9 release) (Table 1). Cases and controls were defined based on ICD10-codes (Table 1). |
|  | b) | Participants: Give the eligibility criteria, and the sources and methods of selection of participants. Report the sample size, and whether any power or sample size calculations were carried out prior to the main analysis | 11-12 | (>80%) Power calculations were calculated (Table S4).  Exposure: The comprehensive summary statistics of genetic influence on human serum metabolites in the TwinsUK and KORA studies provide extensive data for GWAS on the human metabolome. The dataset includes genome-wide genotyping data from 7,824 European participants, and a total of 486 metabolite concentrations were tested in the GWAS Table S1).  Outcome: Table 1 |
|  | c) | Describe measurement, quality control and selection of genetic variants | 12 | The lowest F-statistic value found in validity testing was 17.64, exceeding the threshold of 10, indicating a negligible probability of encountering weak instrument bias (Table S5). Only independent SNPs (r^2^= 0.01; distance = 5,000 kb), strongly associated (*P* ≤ 1 × 10^-5^) with the blood level of each metabolite, were used in the primary analyses (Table S1). |
|  | d) | For each exposure, outcome, and other relevant variables, describe methods of assessment and diagnostic criteria for diseases | 7-8  10 | Exposure: A total of 486 metabolites (Table S1).  Outcome: Cases and controls were defined based on ICD10-codes (Table 1). |
|  | e) | Provide details of ethics committee approval and participant informed consent, if relevant | 9-10 | The studies in these consortia obtained approval from local research ethics committees and institutional review boards, and all participants provided written informed consent. |
| 5 | Assumptions | Explicitly state the three core IV assumptions for the main analysis (relevance, independence and exclusion restriction) as well assumptions for any additional or sensitivity analysis | 7  10-11 | Three necessary assumptions were explained.  We employed MR-Egger method, weighted median analysis, and MR-PRESSO test as sensitivity analysis methods |
| 6 | Statistical methods: main analysis | Describe statistical methods and statistics used |  |  |
|  | a) | Describe how quantitative variables were handled in the analyses (i.e., scale, units, model) | NA |  |
|  | b) | Describe how genetic variants were handled in the analyses and, if applicable, how their weights were selected | NA |  |
|  | c) | Describe the MR estimator (e.g. two-stage least squares, Wald ratio) and related statistics. Detail the included covariates and, in case of two-sample MR, whether the same covariate set was used for adjustment in the two samples | 10  NA | The fixed-effects or random-effects IVW method  Table S1 and 2 for exposure. |
|  | d) | Explain how missing data were addressed | 8 | We excluded 34 metabolite traits that could not be assigned IVs, leaving us with a subset of 452 serum metabolites for further analysis. |
|  | e) | If applicable, indicate how multiple testing was addressed | 11 | A p-value less than 0.05 was considered a nominal association. False Discovery Rate (FDR) correction was employed to control for false positives in multiple tests. Associations were considered statistically significant if the estimated causal effect of a given metabolite had an FDR value of < 0.05. |
| 7 | Assessment of assumptions | Describe any methods or prior knowledge used to assess the assumptions or justify their validity | 8 | Only independent SNPs (r^2^= 0.01; distance = 5,000 kb), strongly associated (*P* ≤ 1 × 10^-5^) with the blood level of each serum metabolites, were used (Table S1 ). |
| 8 | Sensitivity analyses and additional analyses | Describe any sensitivity analyses or additional analyses performed (e.g. comparison of effect estimates from different approaches, independent replication, bias analytic techniques, validation of instruments, simulations) | 17-18  18  12,18  18  18 | We conducted sensitivity analyses to account for pleiotropy for genetic instruments with ≥ 3 variants: MR Egger, weighted median and MR-PRESSO (Table S2 and 4).  We conducted the Cochran's Q statistical test to assess heterogeneity (Table S1 and 4).  For exposures that were significantly associated with an outcome, we checked each exposure SNP in Phenoscanner (Table S4) to assess whether the association could be attributed to pleiotropy. We also performed the MR-PRESSO global test to evaluate pleiotropy (Table S2)  We performed leave-one-out analyses to examine the robustness of the IVW estimates and whether any specific SNP drove the association (Additional File 2)  A reverse MR analysis was conducted to explore potential causal effects of stroke phenotypes on serum metabolites. |
| 9 | Software and pre-registration |  |  |  |
|  | a) | Name statistical software and package(s), including version and settings used | 11  11  12 | The statistical analyses were performed using R software version 4.2.3. The MR analyses were performed using the TwoSampleMR package and the MRPRESSO package.  (>80%) Power calculations were calculated using an online tool available at <http://cnsgenomics.com/shiny/mRnd/>  Phenoscanner, available at <http://www.phenoscanner.medschl.cam.ac.uk/>, was used to evaluate whether the association could be due to pleiotropy. |
|  | b) | State whether the study protocol and details were pre-registered (as well as when and where) |  | No. |
|  | Results |  |  |  |
| 10 | Descriptive data |  |  |  |
|  | a) | Report the numbers of individuals at each stage of included studies and reasons for exclusion. Consider use of a flow diagram |  | Exposure: Table S1 and Figure1  Outcome: Table 1. |
|  | b) | Report summary statistics for phenotypic exposure(s), outcome(s), and other relevant variables (e.g. means, SDs, proportions) |  | Exposure: Table S1. Outcomes: Table 1 |
|  | c) | If the data sources include meta-analyses of previous studies, provide the assessments of heterogeneity across these studies |  | NA |
|  | d) | For two-sample MR:  i.  Provide justification of the similarity of the genetic variant-exposure associations between the exposure and outcome samples  ii.  Provide information on the number of individuals who overlap between the exposure and outcome studies |  | i: We used different data sources for exposures and outcomes. We calculated the Cochran's Q-test for heterogeneity to assess heterogeneity across the cohorts and found minimal heterogeneity for the included variants for the outcomes (Table S1)  ii: Because we used summary-level statistics, we couldn't identify individuals who were common to both the exposure and outcome datasets. |
| 11 | Main results |  |  |  |
|  | a) | Report the associations between genetic variant and exposure, and between genetic variant and outcome, preferably on an interpretable scale |  | Figure 3, Table S1 and S4 for MR analysis, using r2 < 0.01 within 5,000 kb windows and *P* ≤ 1E-05. |
|  | b) | Report MR estimates of the relationship between exposure and outcome, and the measures of uncertainty from the MR analysis, on an interpretable scale, such as odds ratio or relative risk per SD difference |  | OR |
|  | c) | If relevant, consider translating estimates of relative risk into absolute risk for a meaningful time period | NA |  |
|  | d) | Consider plots to visualize results (e.g. forest plot, scatterplot of associations between genetic variants and outcome versus between genetic variants and exposure) |  | Figure 3 and 4. |
| 12 | Assessment of assumptions |  |  |  |
|  | a) | Report the assessment of the validity of the assumptions | 17-18 | Methods to assess the robustness of MR findings: MR Egger, weighted median, MRPRESSO, Cochran's Q statistical test, PhenoScanner, leave-one-out analyses and reverse MR analysis. |
|  | b) | Report any additional statistics (e.g., assessments of heterogeneity across genetic variants, such as I2, Q statistic or E-value) | 18 | The I^2^ value and Q-test were used to assess potential heterogeneity and identify outliers in the IVW and MR-Egger analyses. |
| 13 | Sensitivity analyses and additional analyses |  |  |  |
|  | a) | Report any sensitivity analyses to assess the robustness of the main results to violations of the assumptions | 18 | Table S2 and S4 for the MR analyses. |
|  | b) | Report results from other sensitivity analyses or additional analyses |  | Table S4 |
|  | c) | Report any assessment of direction of causal relationship (e.g., bidirectional MR) | 18 | We conducted a bidirectional two-sample MR analysis using stroke subtypes as the exposure and blood levels of metabolites as the outcome. |
|  | d) | When relevant, report and compare with estimates from non-MR analyses | NA |  |
|  | e) | Consider additional plots to visualize results (e.g., leave-one-out analyses) |  | Leave-one-out results are presented in Additional file 2. |
|  | Disscusion |  |  |  |
| 14 | Key results | Summarize key results with reference to study objectives | 20 | The IVW method revealed 266 causative associations (p < 0.05) between serum metabolites and 11 stroke phenotype traits (Table S2). These associations correspond to 151 specific metabolites (Figure 2). After FDR correction, we observed 25 metabolites with significant causative correlations to stroke and its subtypes (FDR < 0.05). |
| 15 | Limitations | Discuss limitations of the study, taking into account the validity of the IV assumptions, other sources of potential bias, and imprecision. Discuss both direction and magnitude of any potential bias and any efforts to address them | 29-30 |  |
| 16 | Interpretation |  |  |  |
|  | a) | Meaning: Give a cautious overall interpretation of results in the context of their limitations and in comparison with other studies | 21-28 |  |
|  | b) | Mechanism: Discuss underlying biological mechanisms that could drive a potential causal relationship between the investigated exposure and the outcome, and whether the gene-environment equivalence assumption is reasonable. Use causal language carefully, clarifying that IV estimates may provide causal effects only under certain assumptions | 21-29 | Given the significant role of metabolites in the development and maintenance of the nervous system, it is biologically plausible that certain metabolites may influence the risk of stroke and its subtypes. Based on our findings, this provides early predictive factors for future research on the utility of these biomarkers in blood tests for stroke prevention. |
|  | c) | Clinical relevance: Discuss whether the results have clinical or public policy relevance, and to what extent they inform effect sizes of possible interventions | 28 |  |
| 17 | Generalizability | Discuss the generalizability of the study results (a) to other populations, (b) across other exposure periods/timings, and (c) across other levels of exposure | 28-29 |  |
|  | Other information |  |  |  |
| 18 | Funding | Describe sources of funding and the role of funders in the present study and, if applicable, sources of funding for the databases and original study or studies on which the present study is based | 31 | The present research utilized publicly available summary data, for which no additional ethical approval was required. This study did not receive any funding. |
| 19 | Data and data sharing | Provide the data used to perform all analyses or report where and how the data can be accessed and reference these sources in the article. Provide the statistical code needed to reproduce the results in the article, or report whether the code is publicly accessible and if so, where |  | Exposure: Table S1 for genetic instruments used as exposure.  Table 1 for genetic instruments used as outcome. |
| 20 | Conflicts of Interest | All authors should declare all potential conflicts of interest | 31 | All authors declare that the research was conducted in the absence of any commercial or financial relationships that could be construed as a potential conflict of interest. |
