# Supplementary figures and images for: Assessing the causal effect of genetically predicted metabolites and metabolic pathways on stroke
Source: J Transl Med. 2023 Nov 17;21:822. doi: 10.1186/s12967-023-04677-4 (PMC10655369; doi:10.1186/s12967-023-04677-4)

**Additional file 2: The result for leave-one-out analysis**

AS


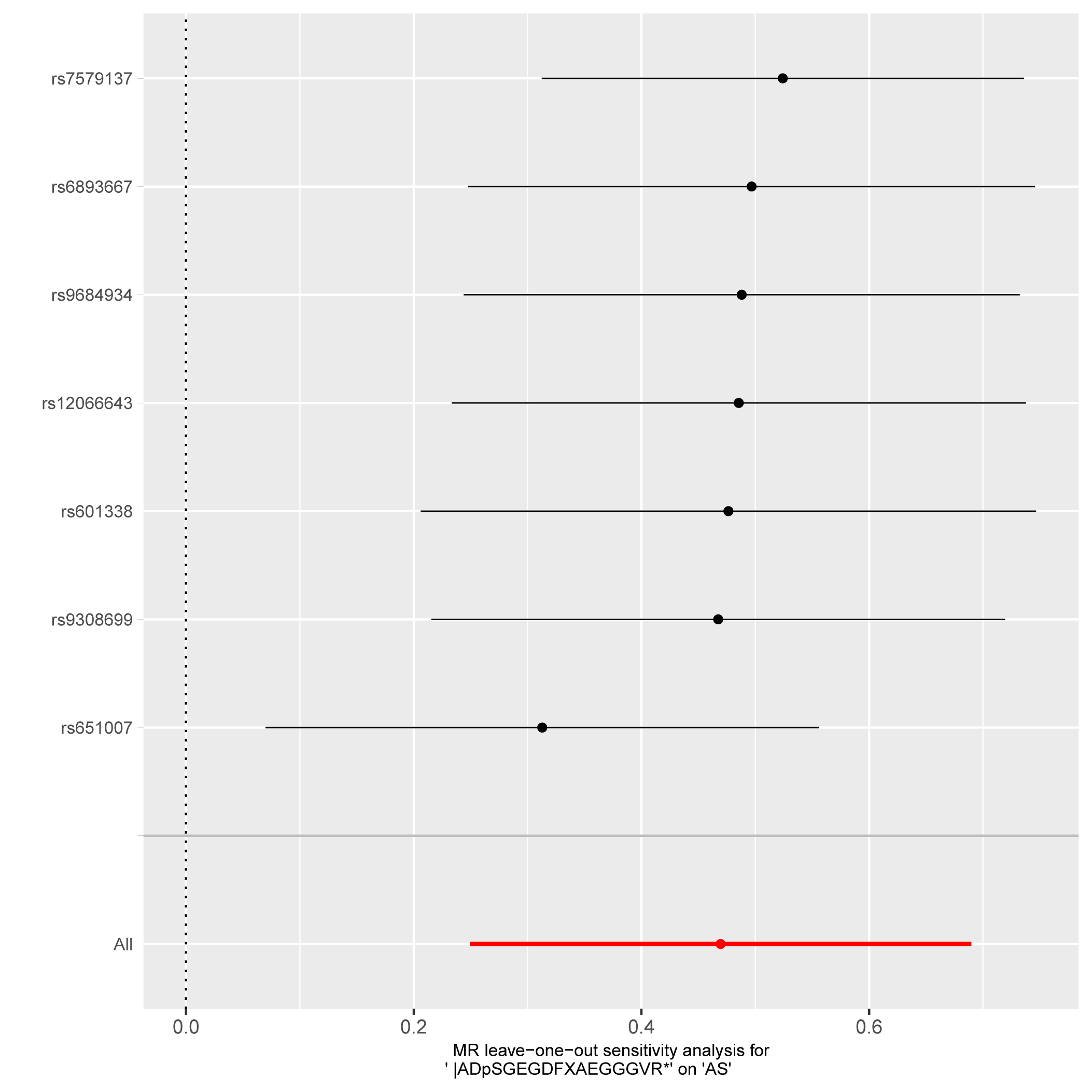


AIS


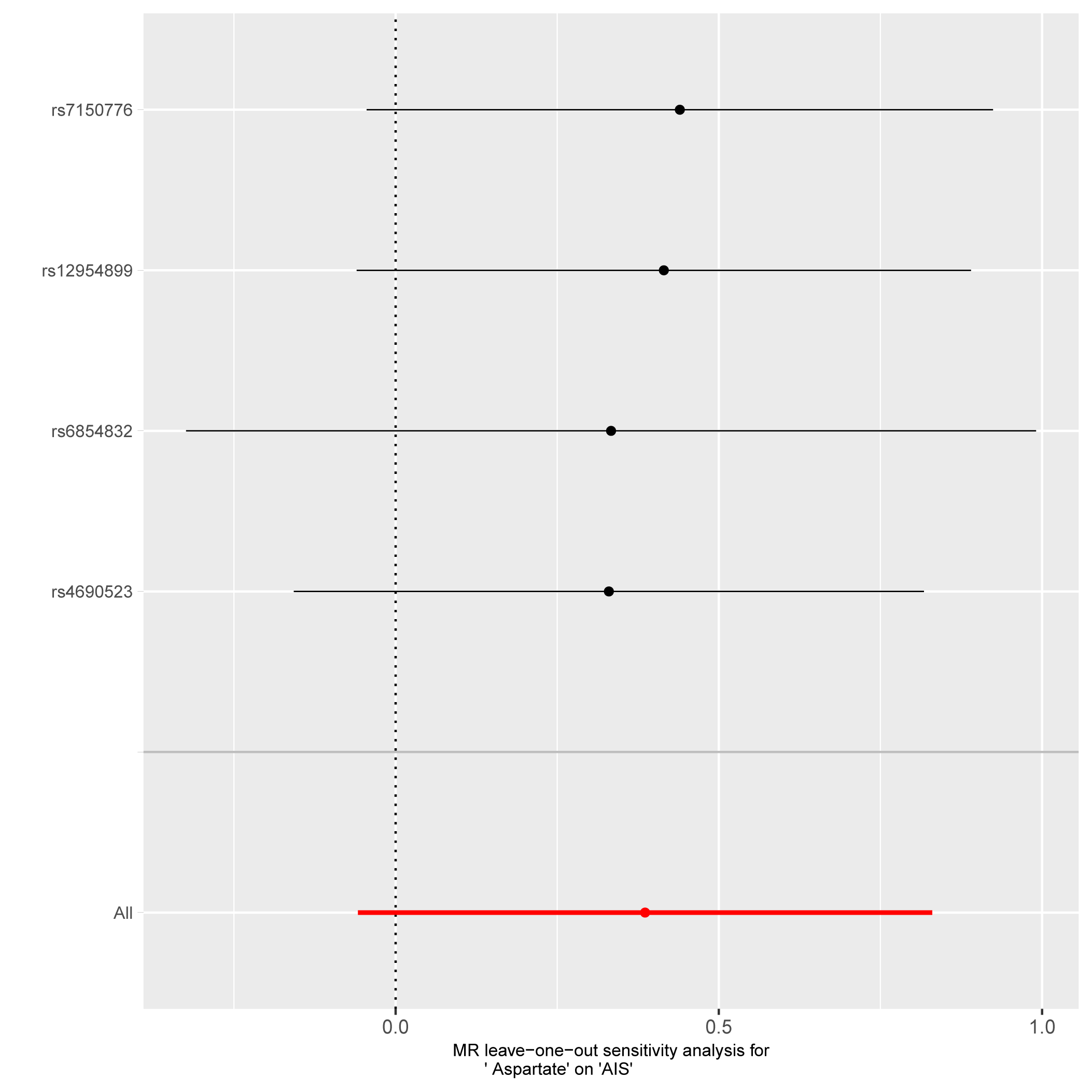

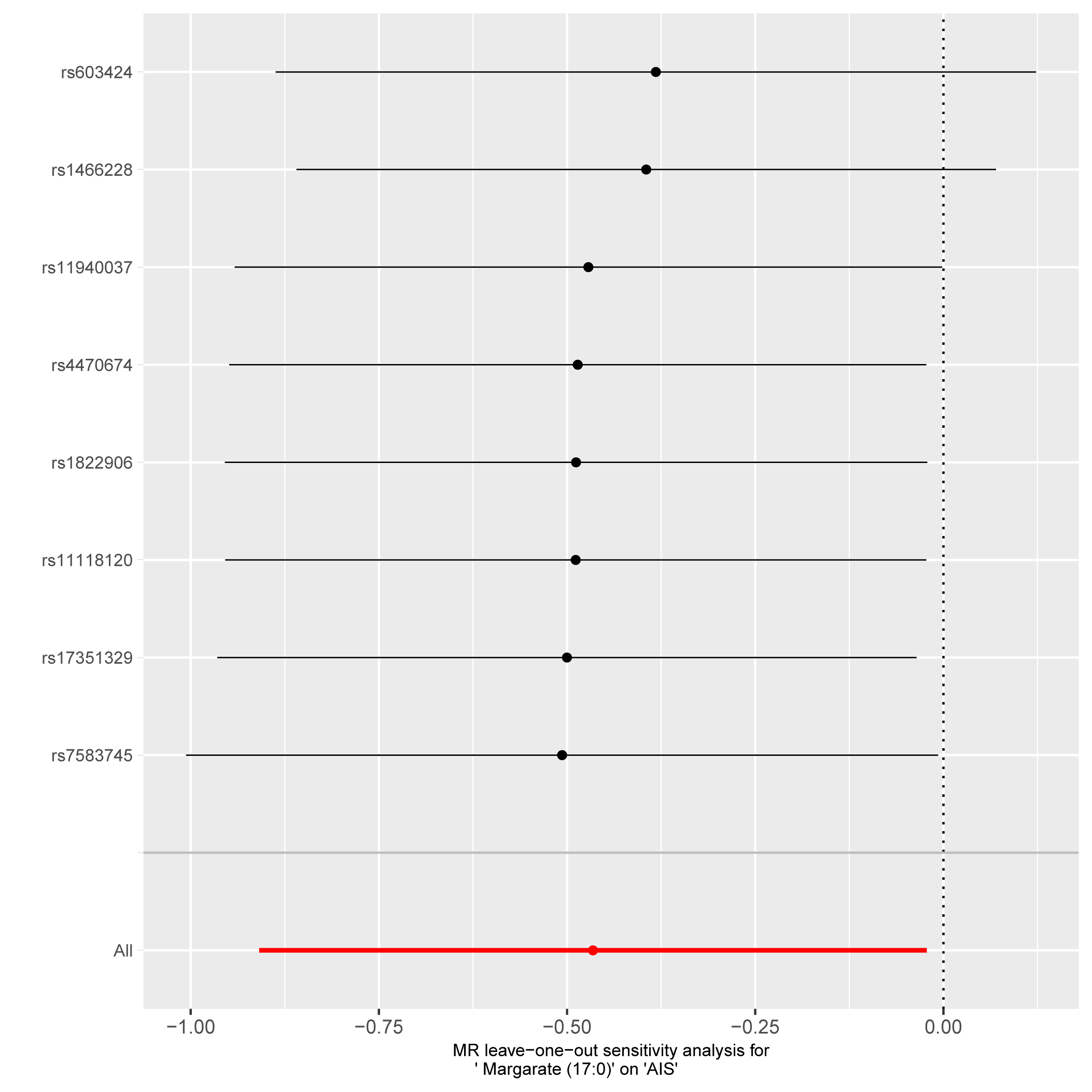


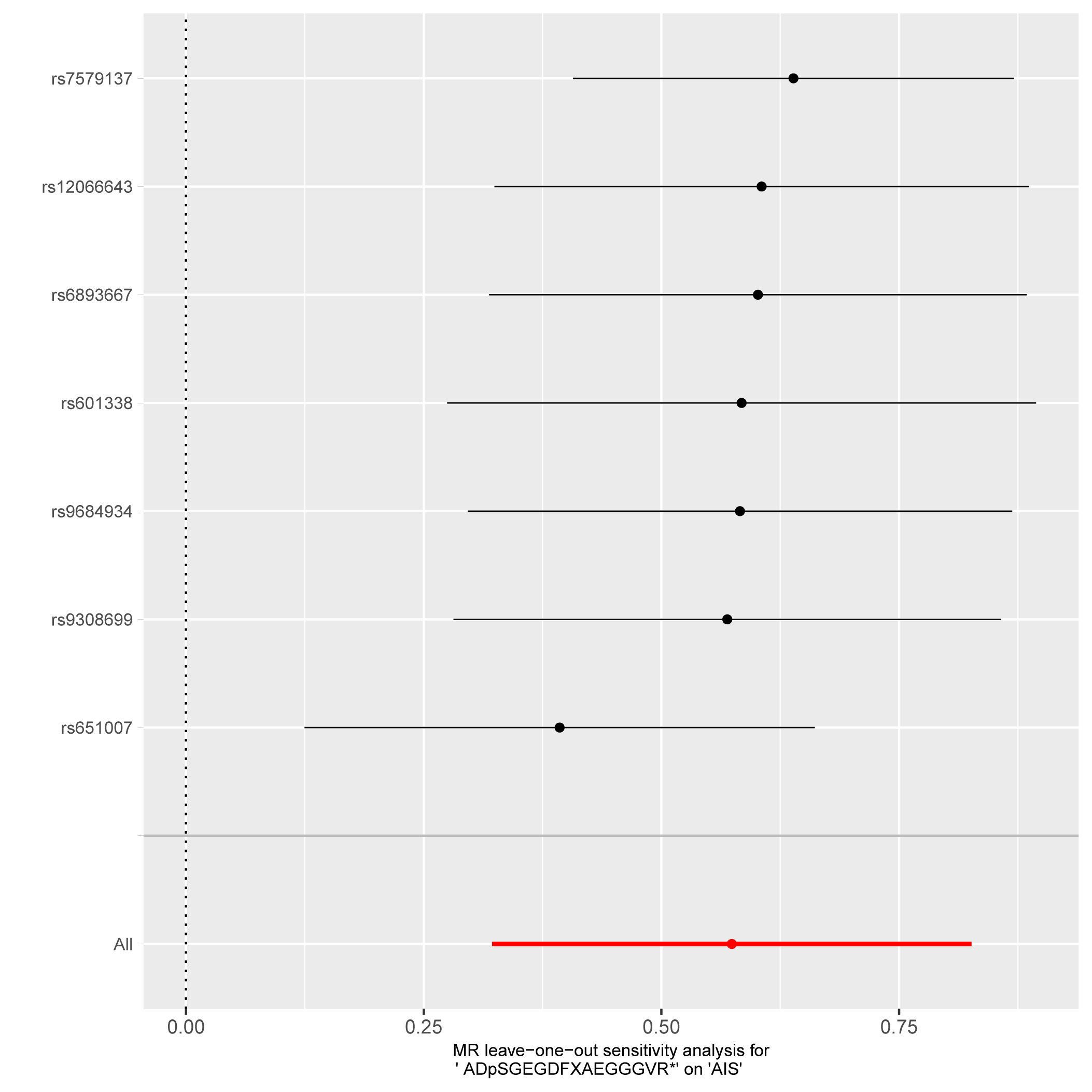


LAS


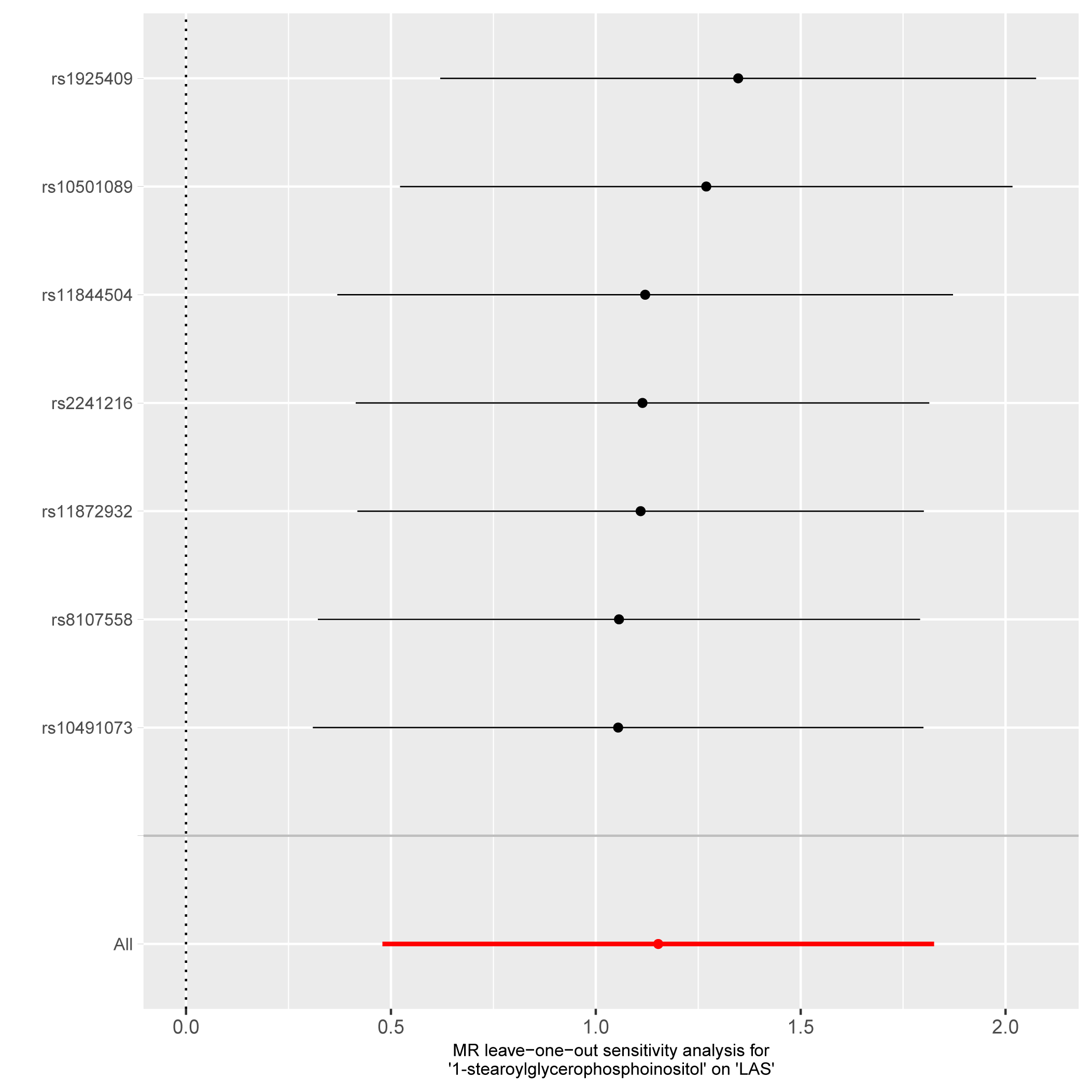

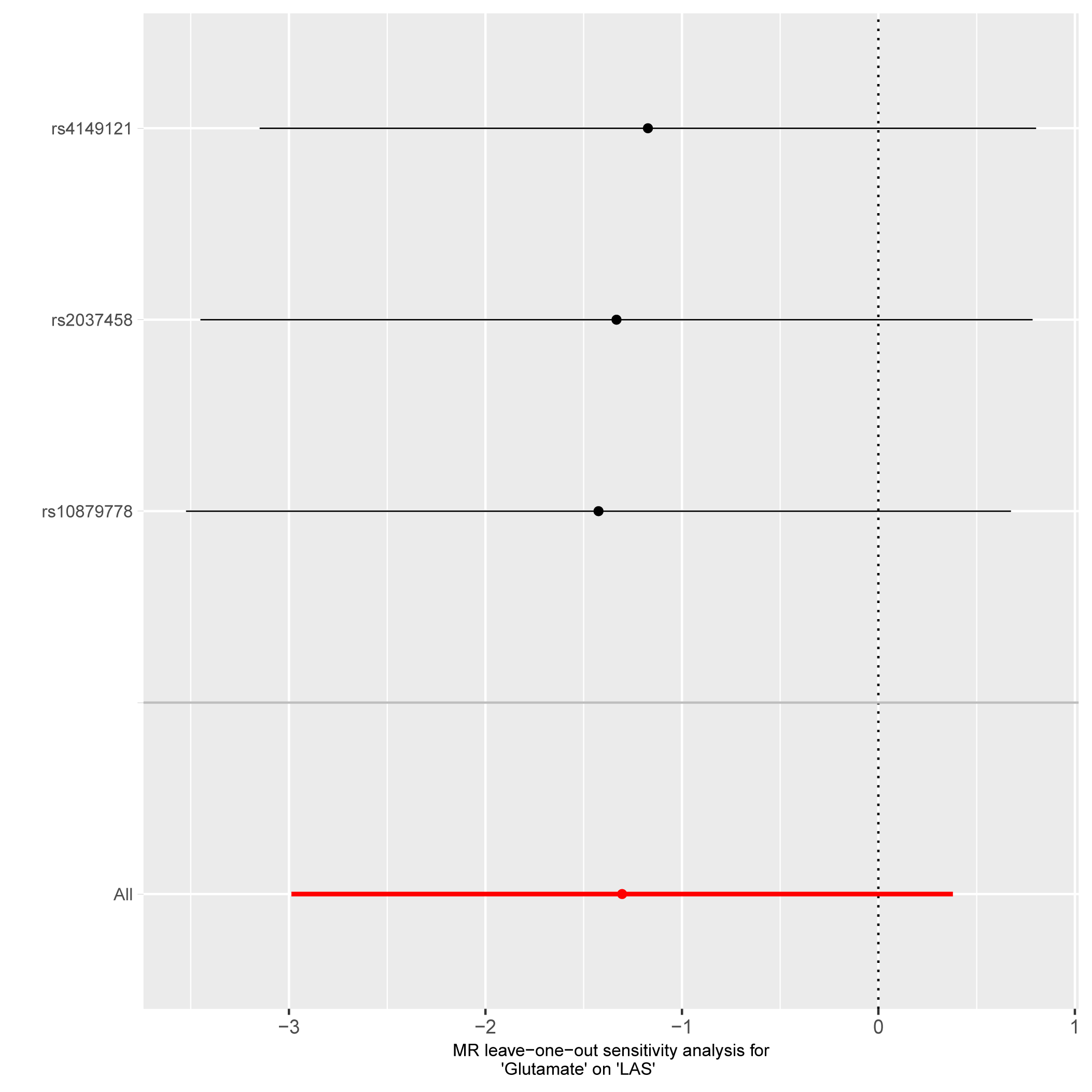

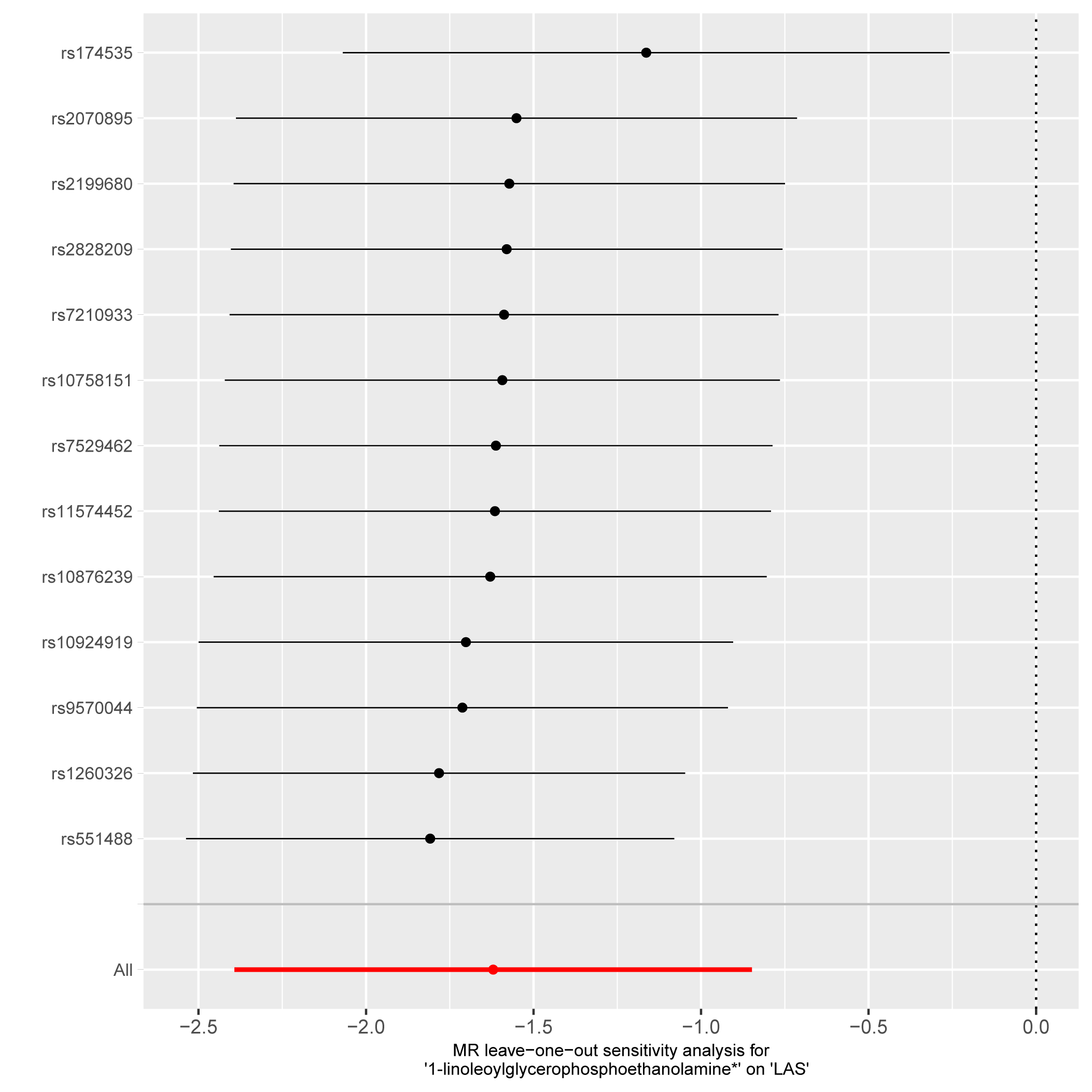

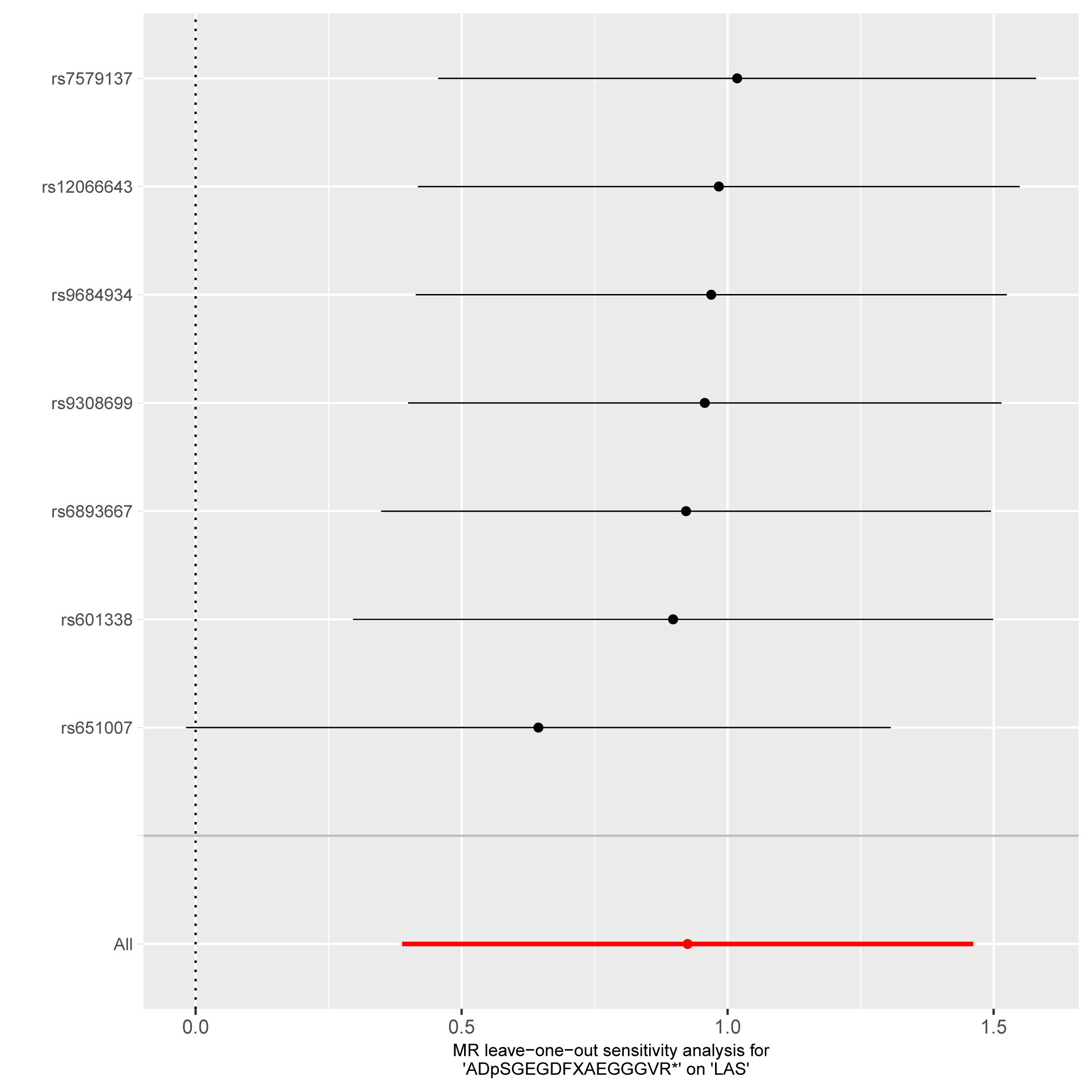

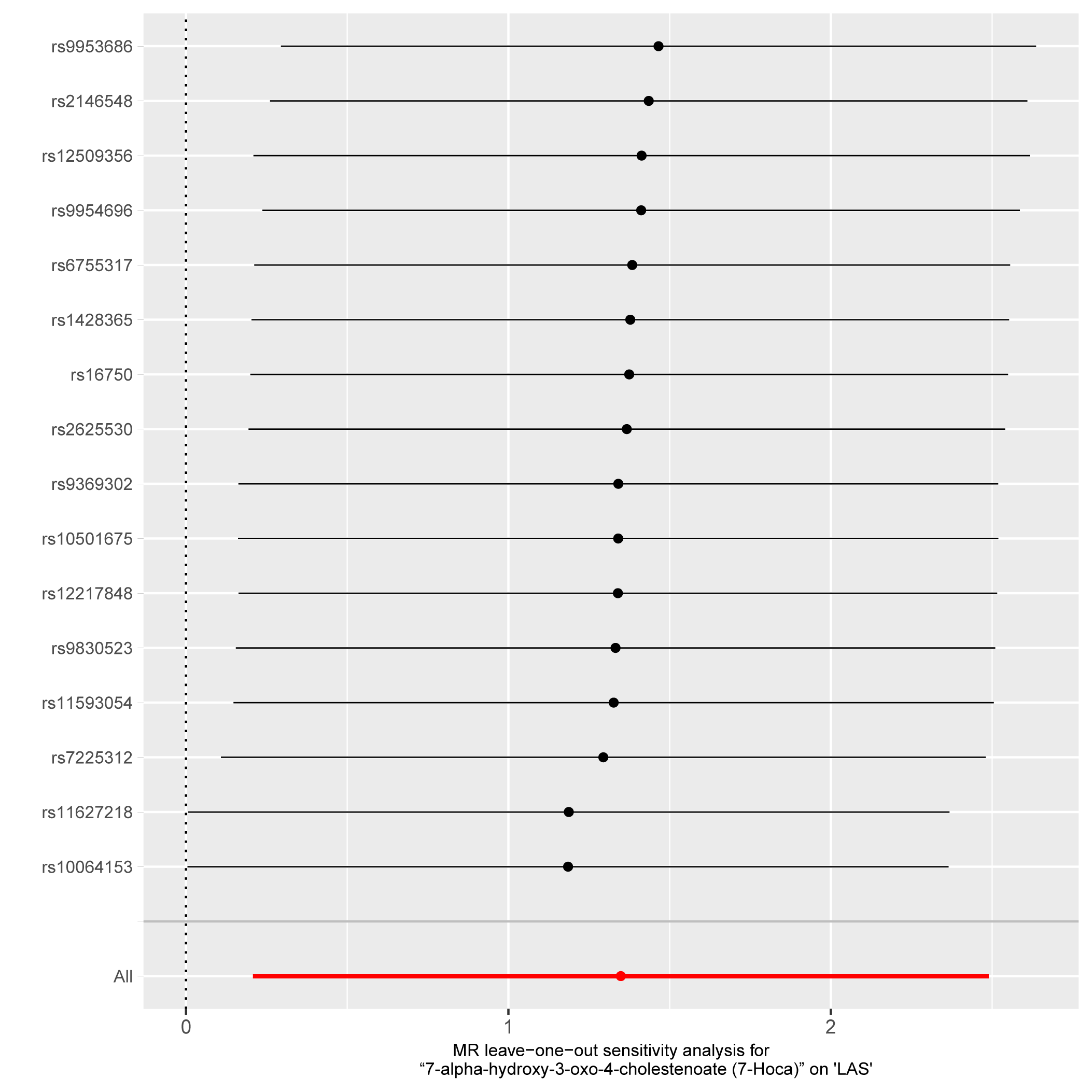


SVS


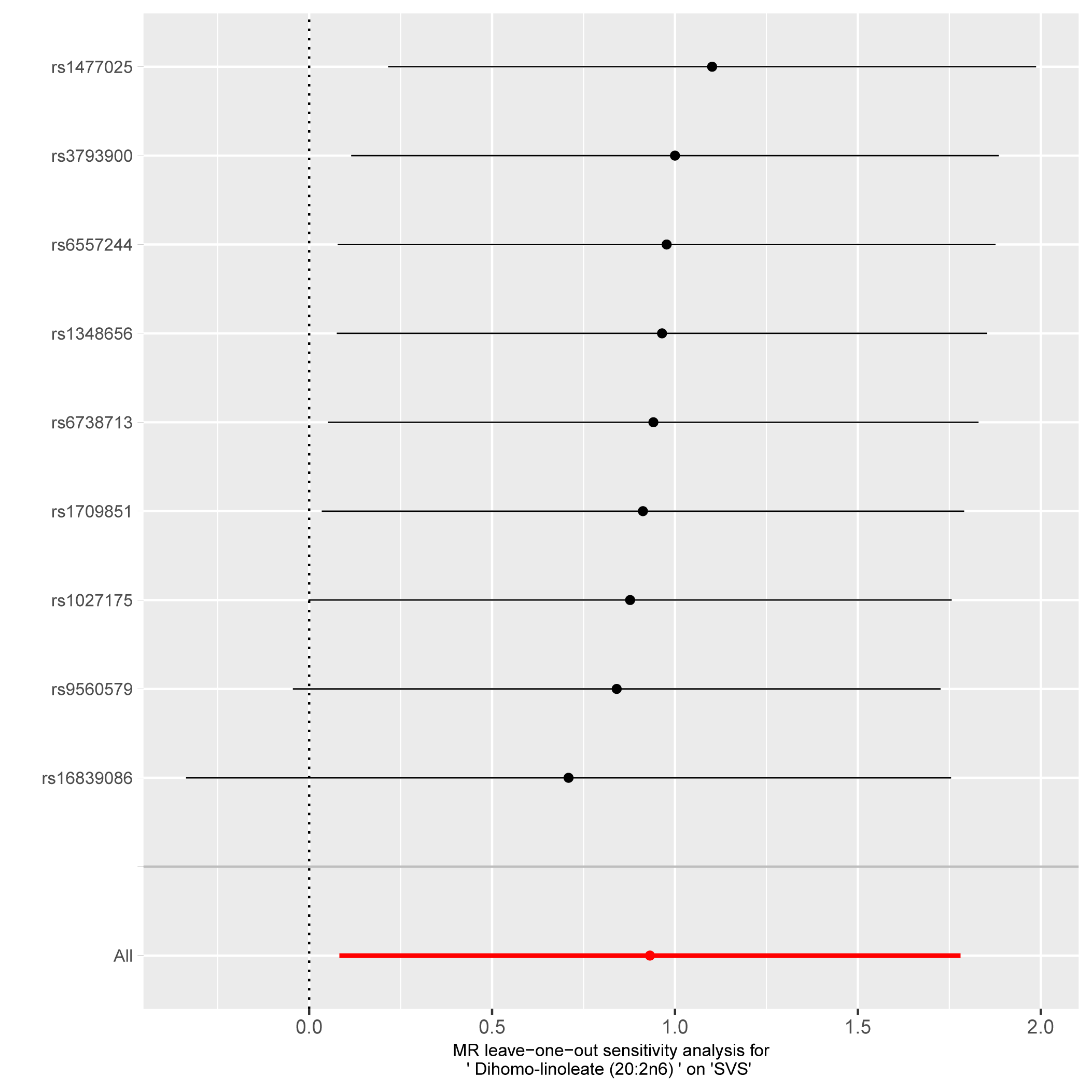

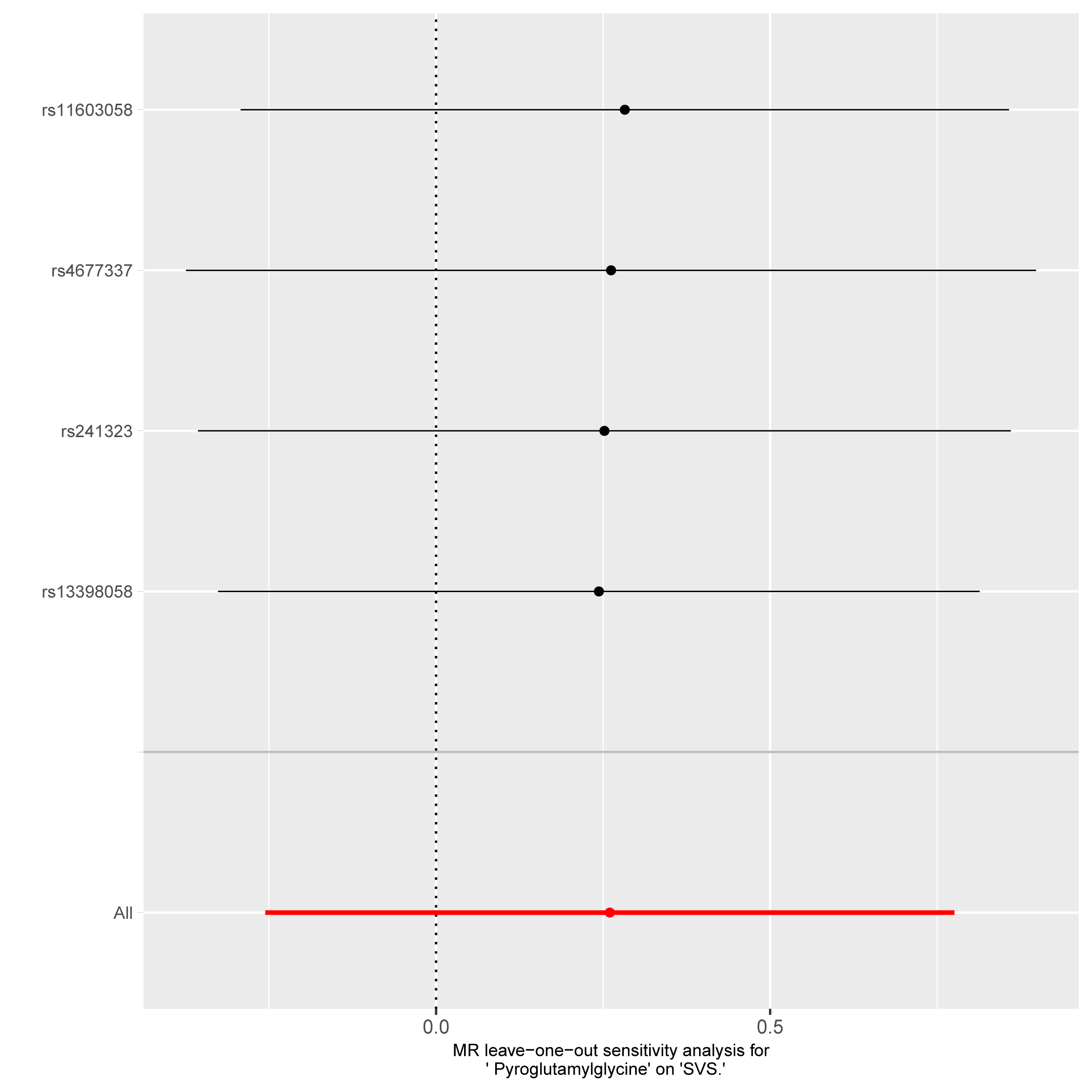

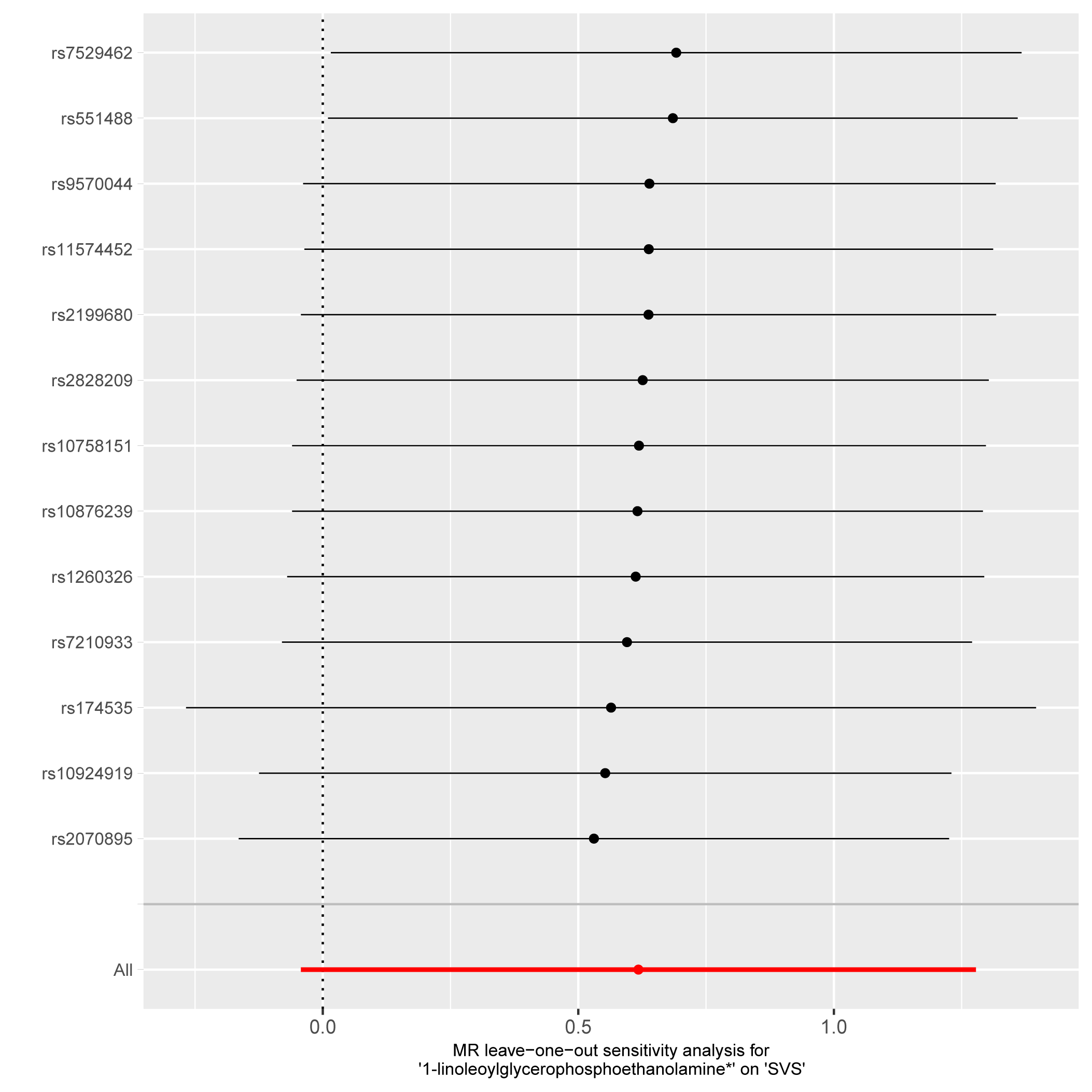

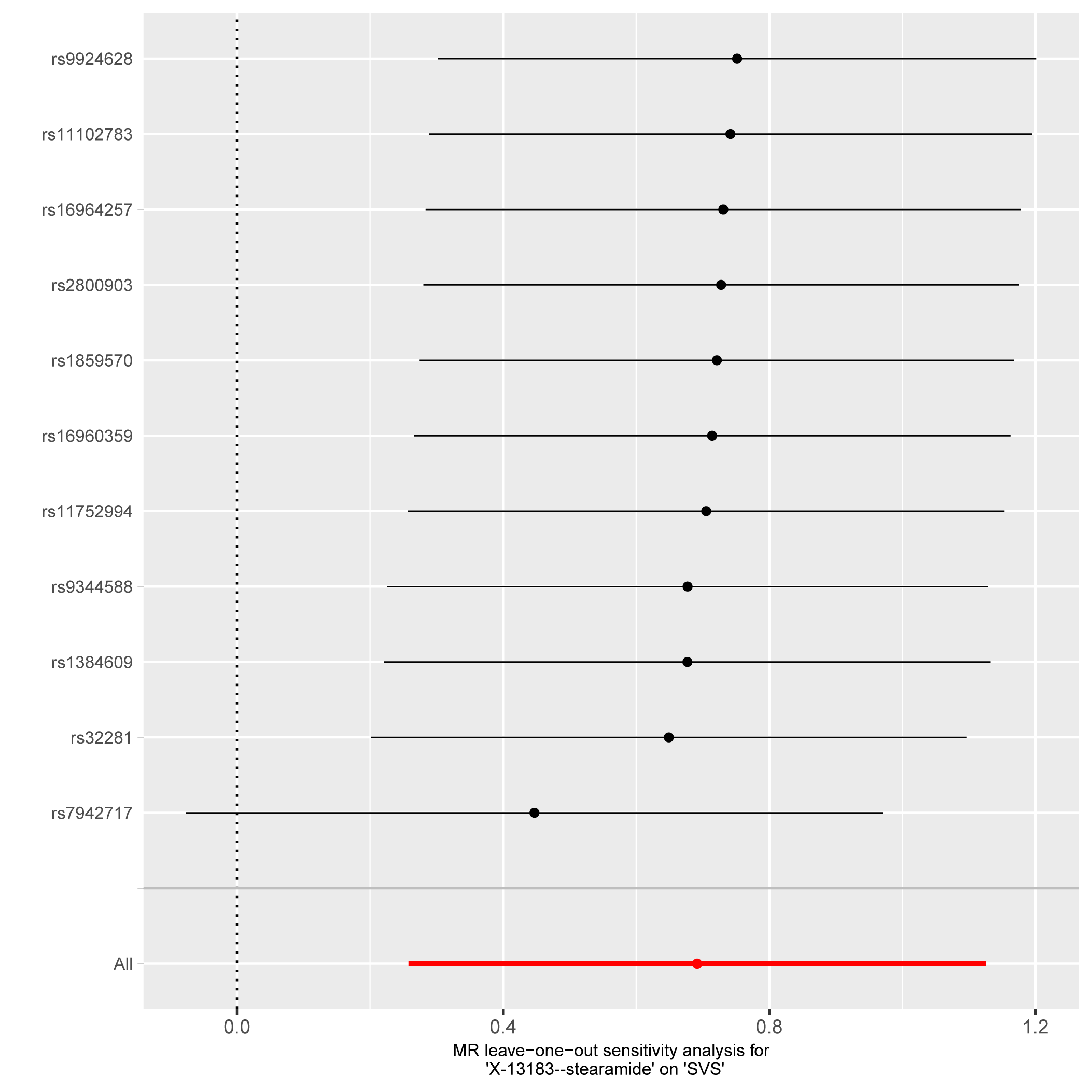

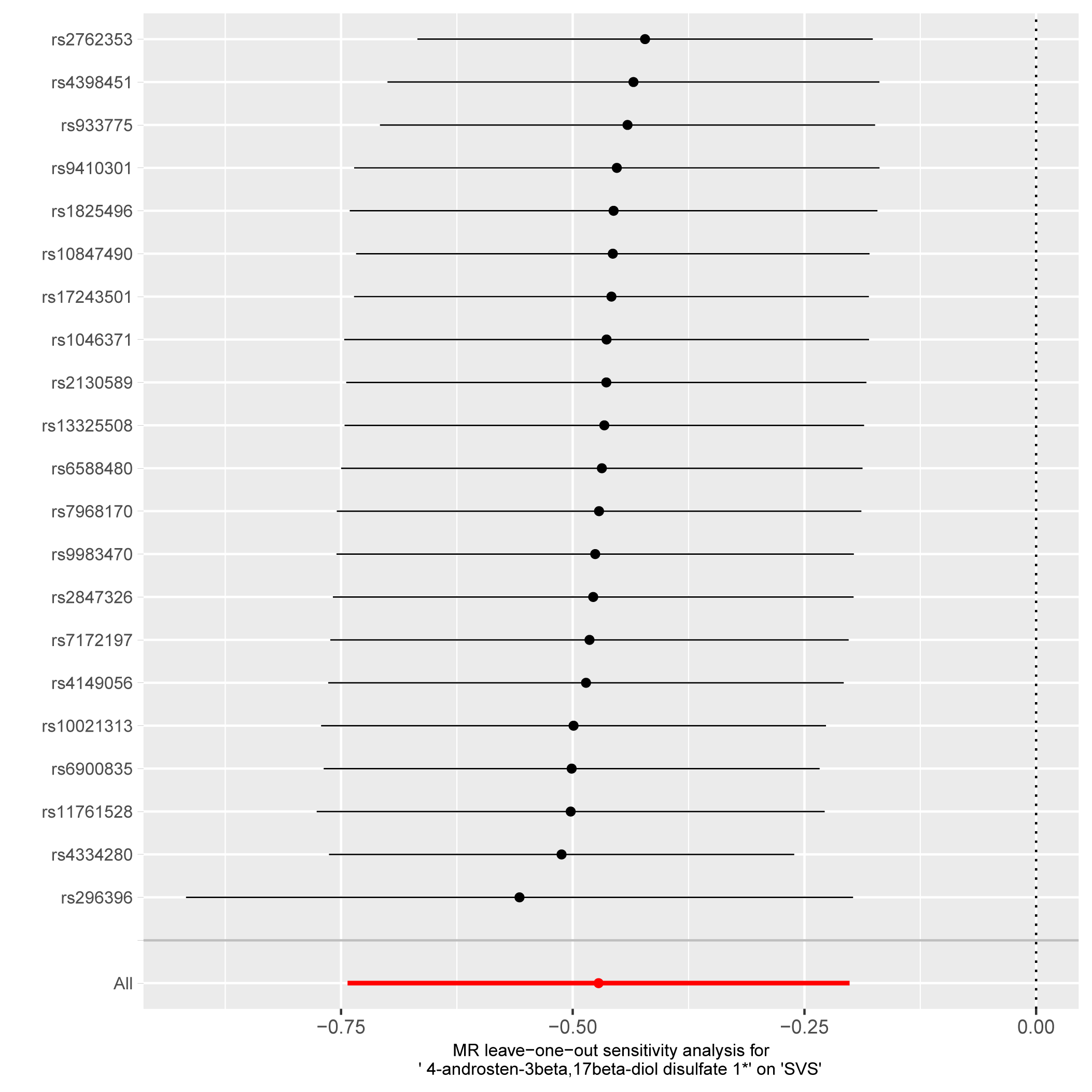


ICH


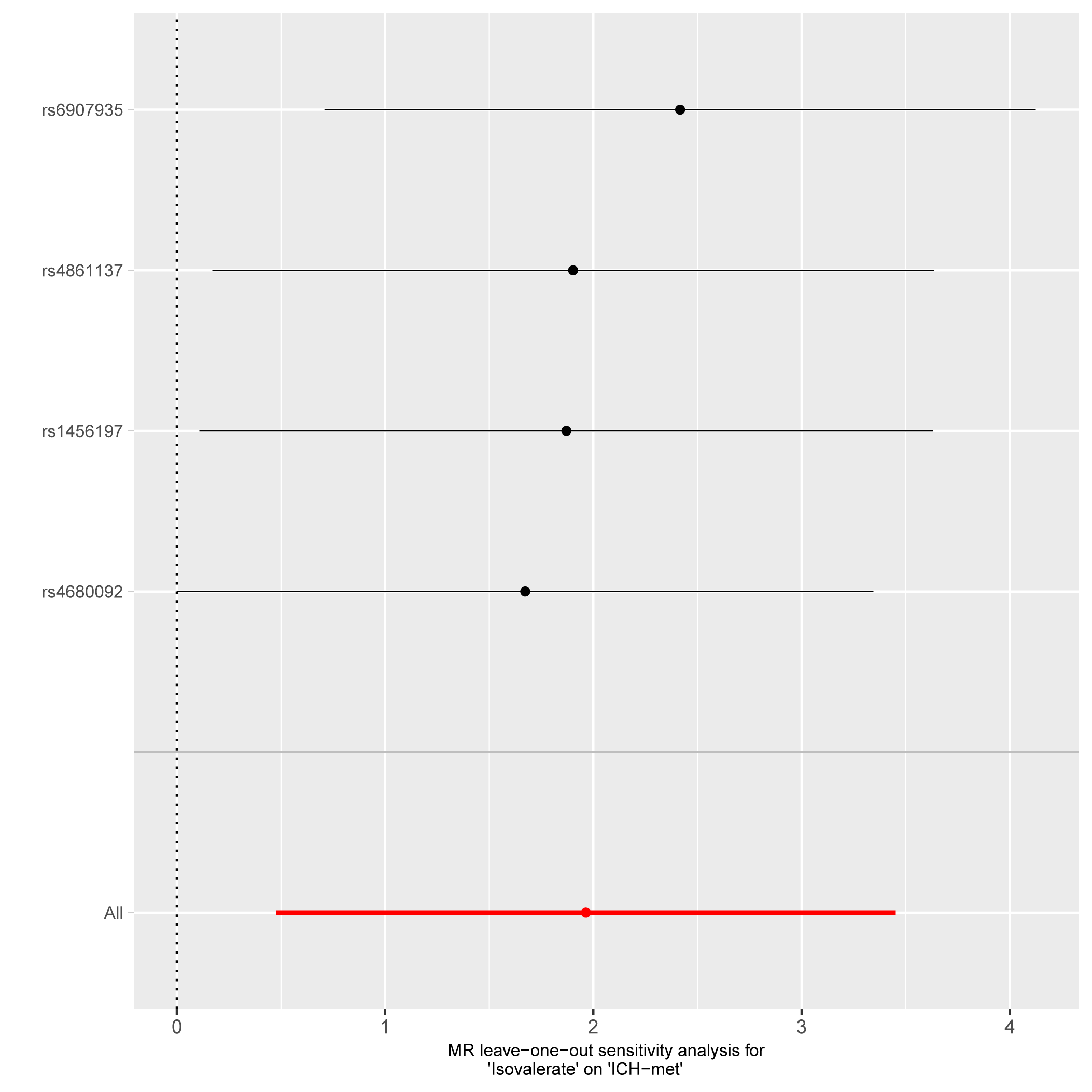


SAH


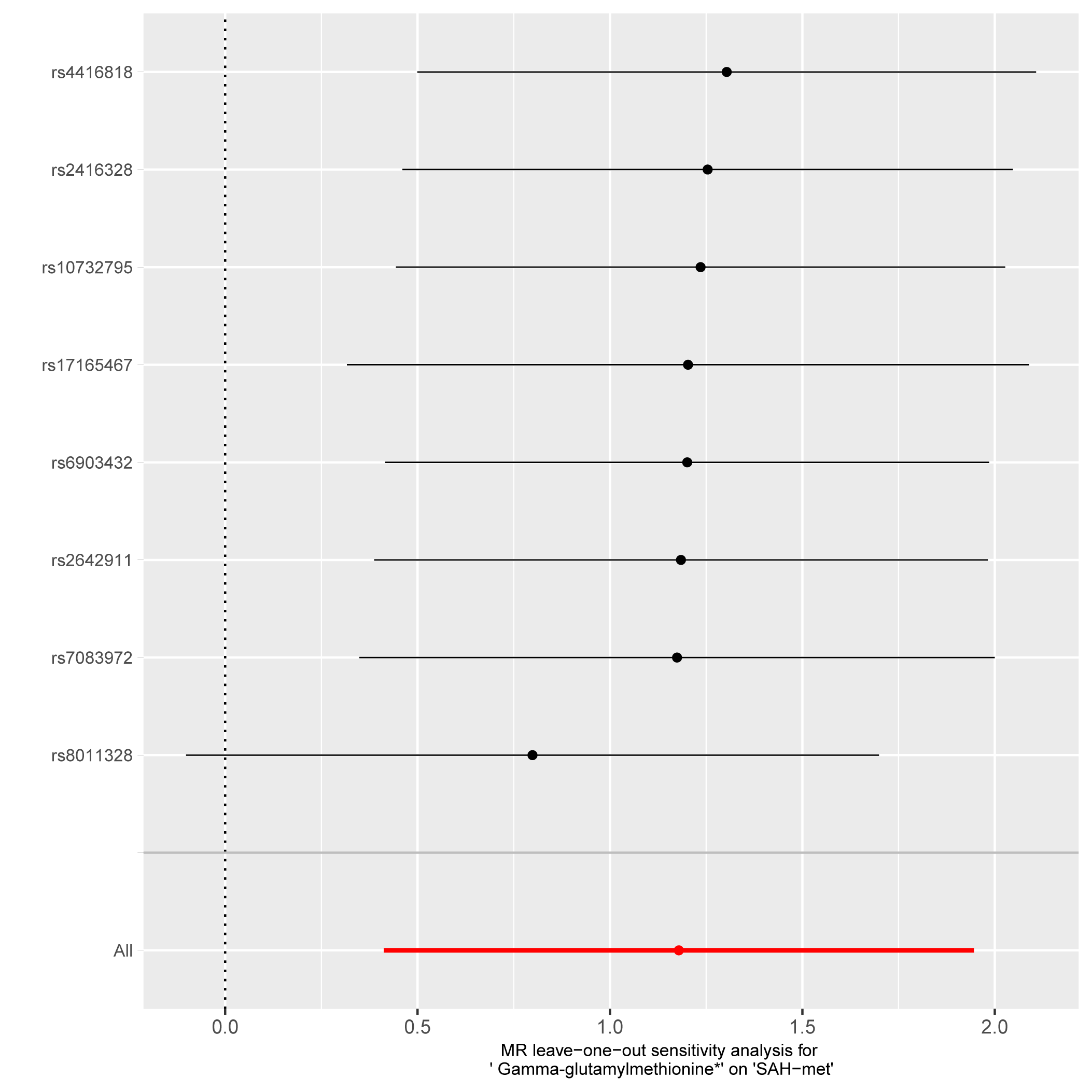

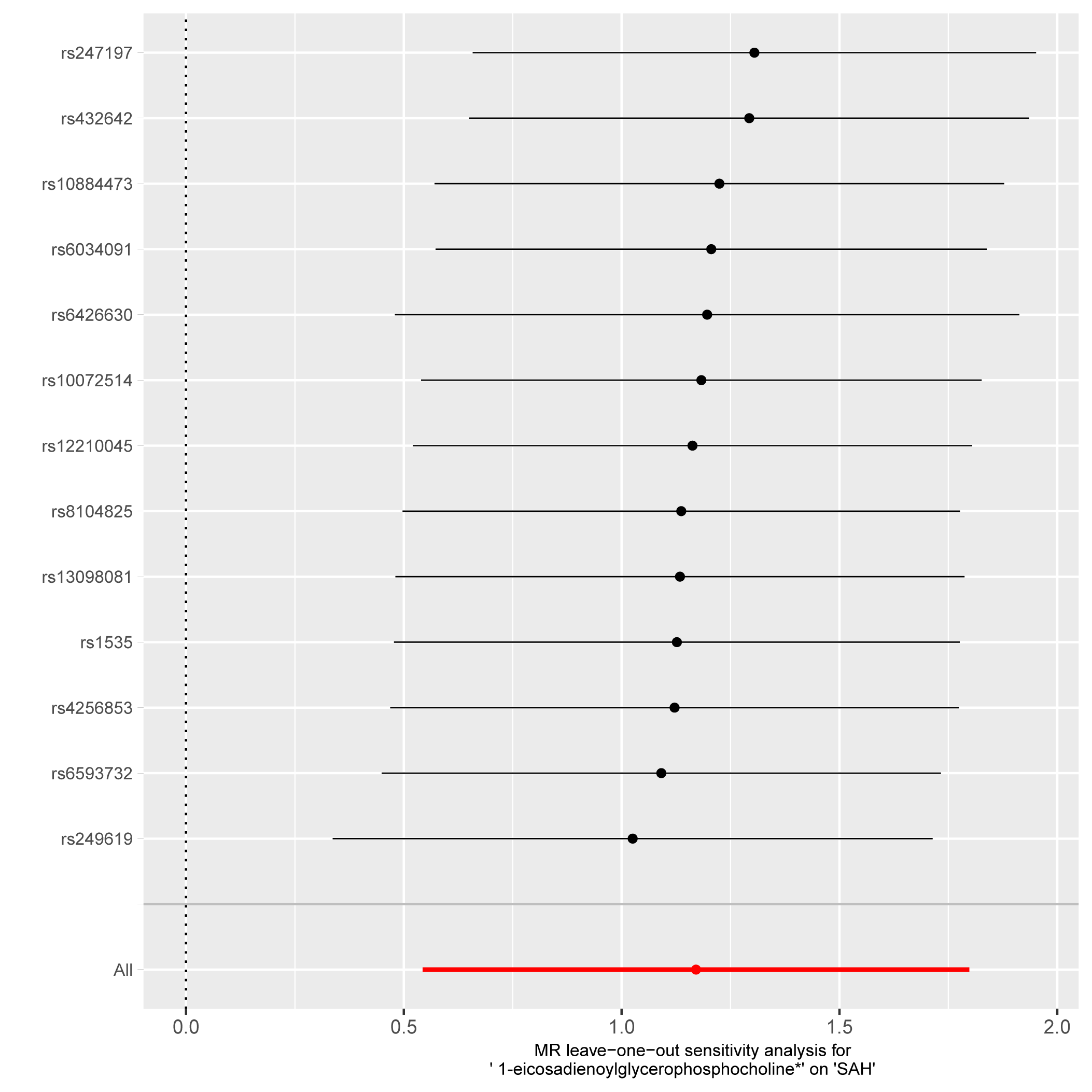


BMB


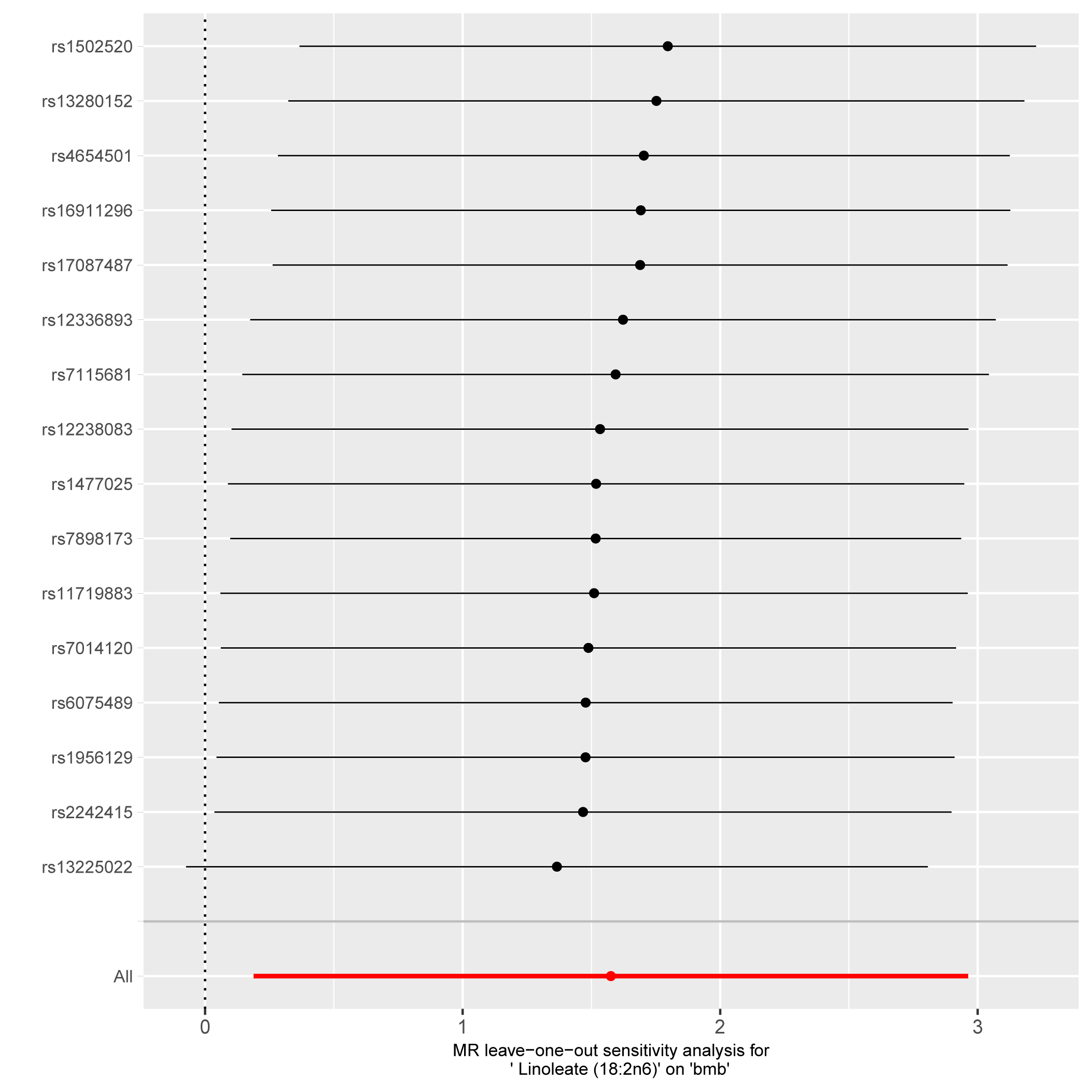

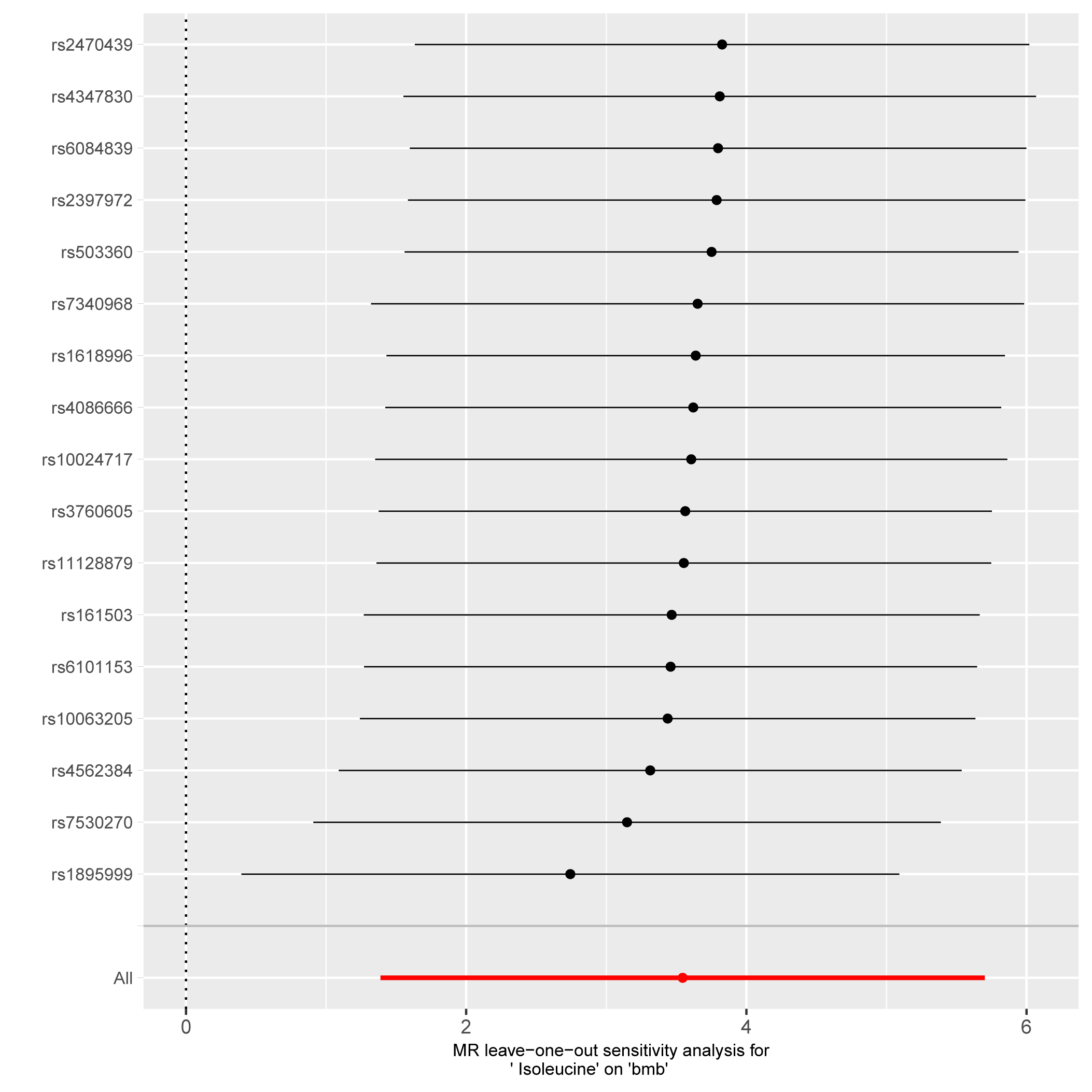

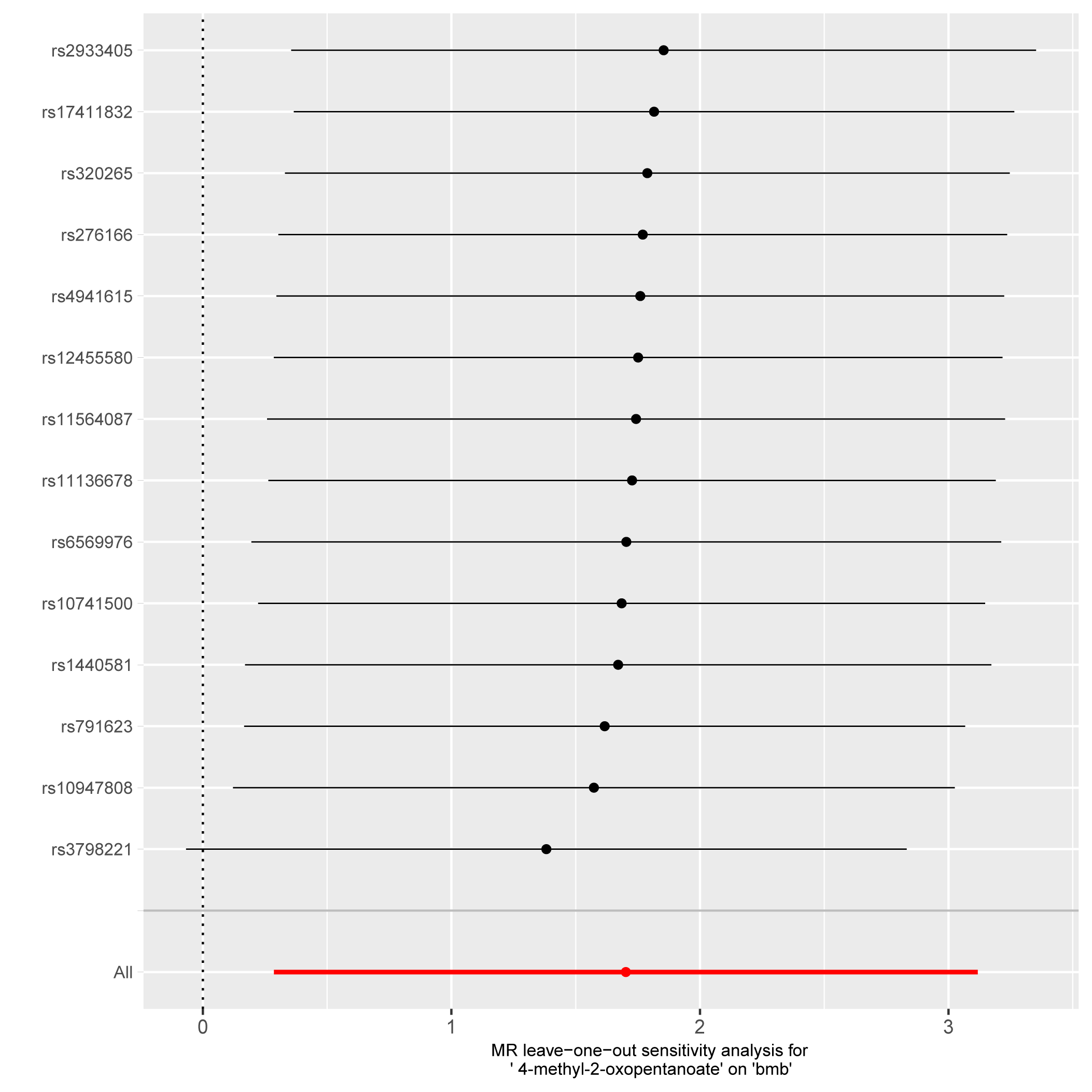

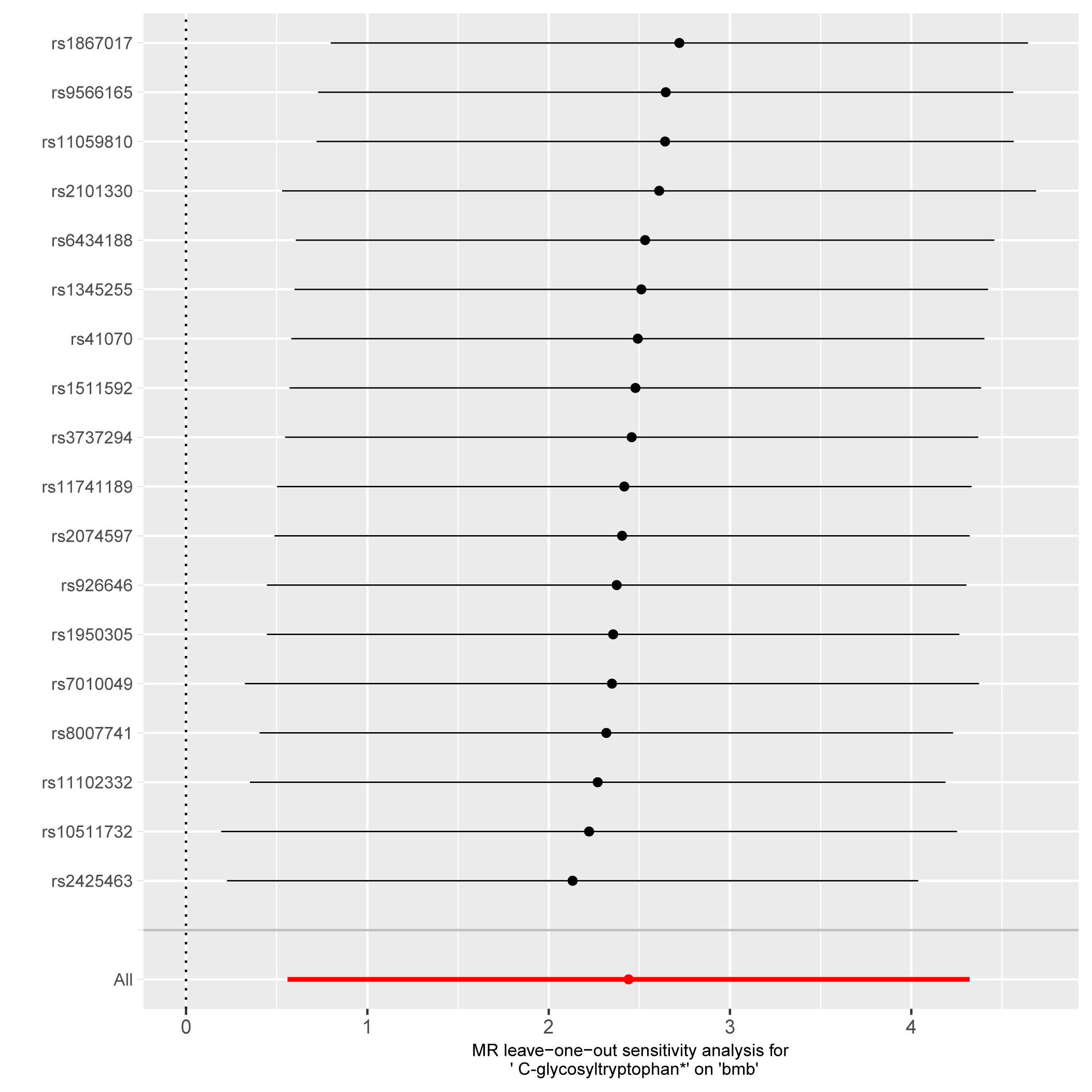

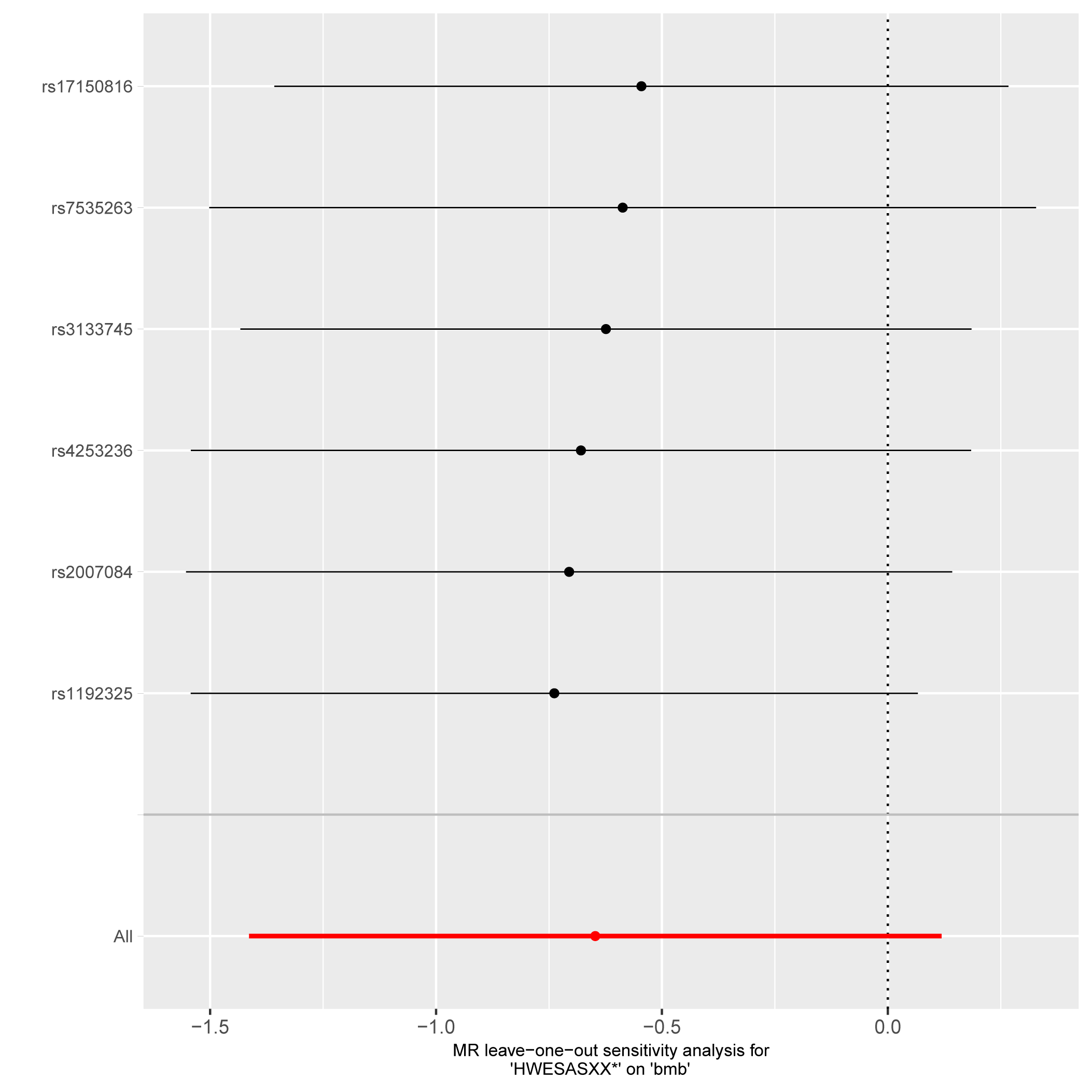

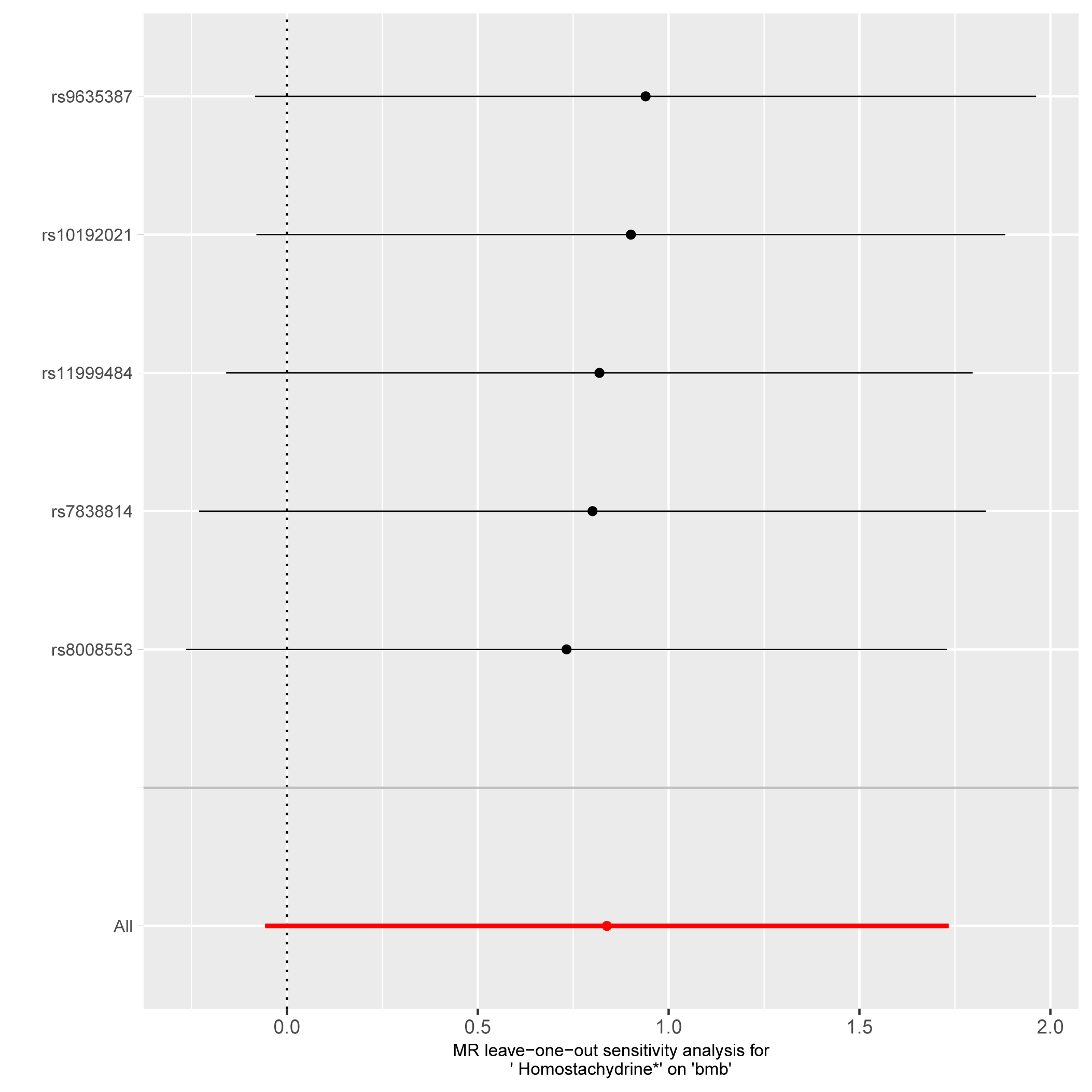


LS


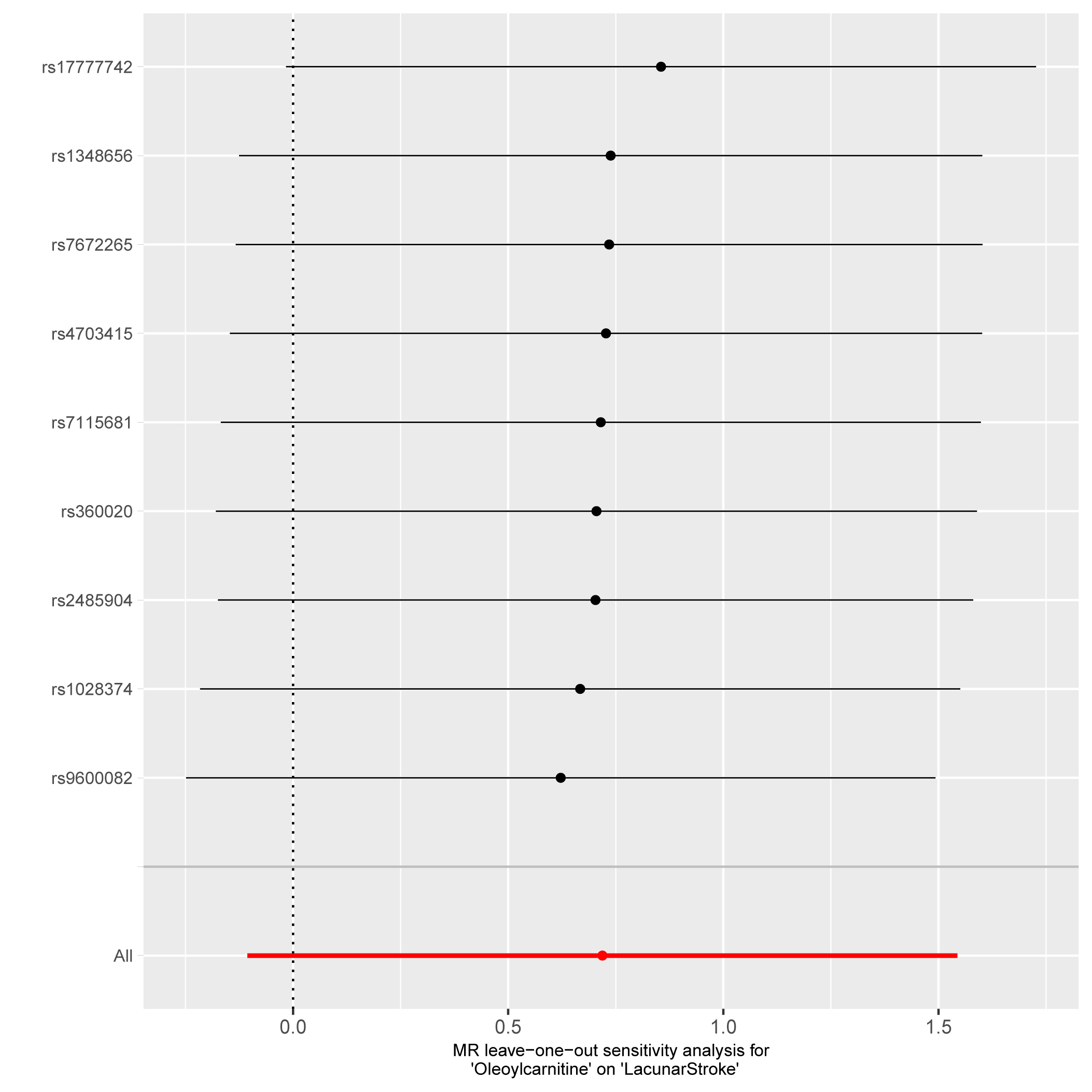


WMH


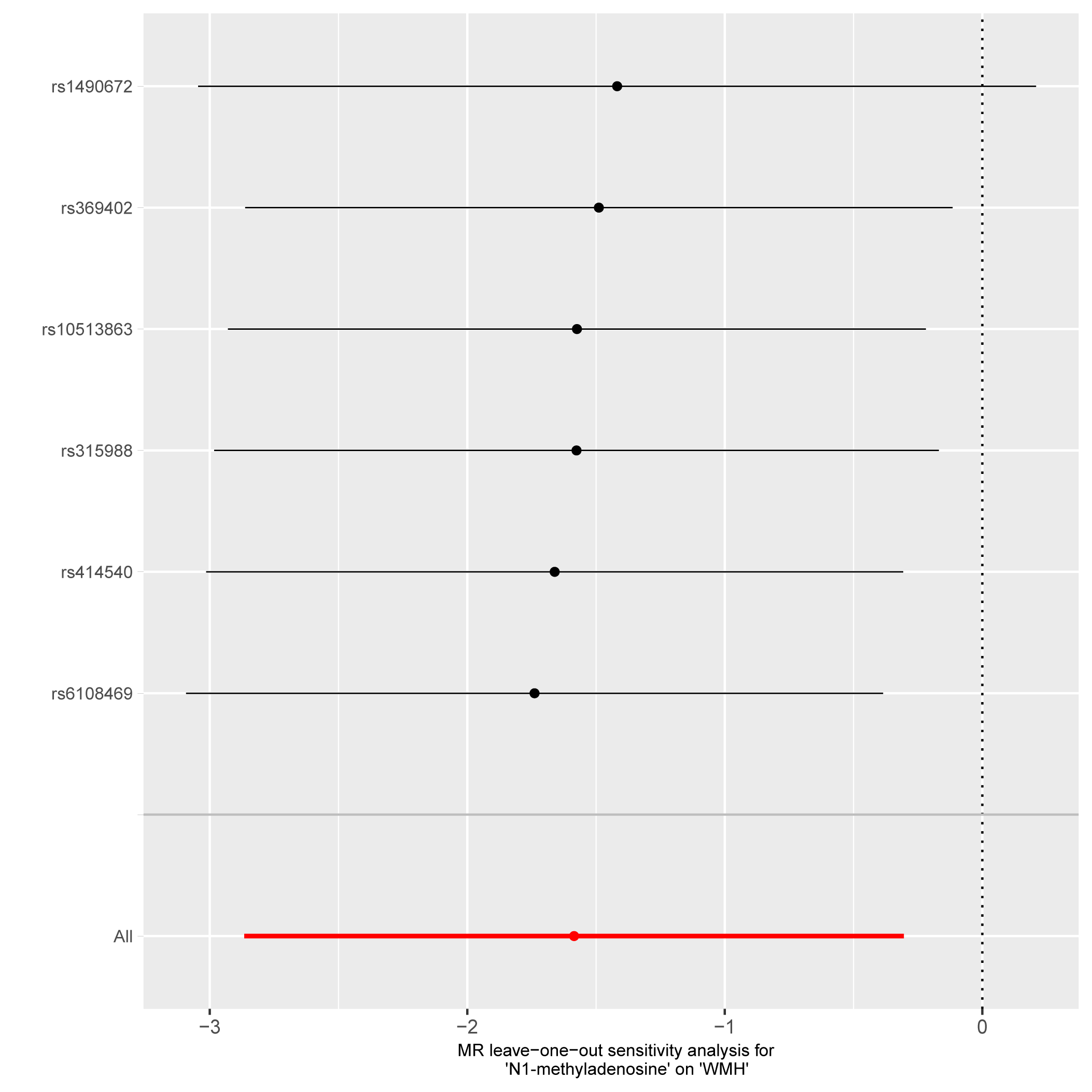

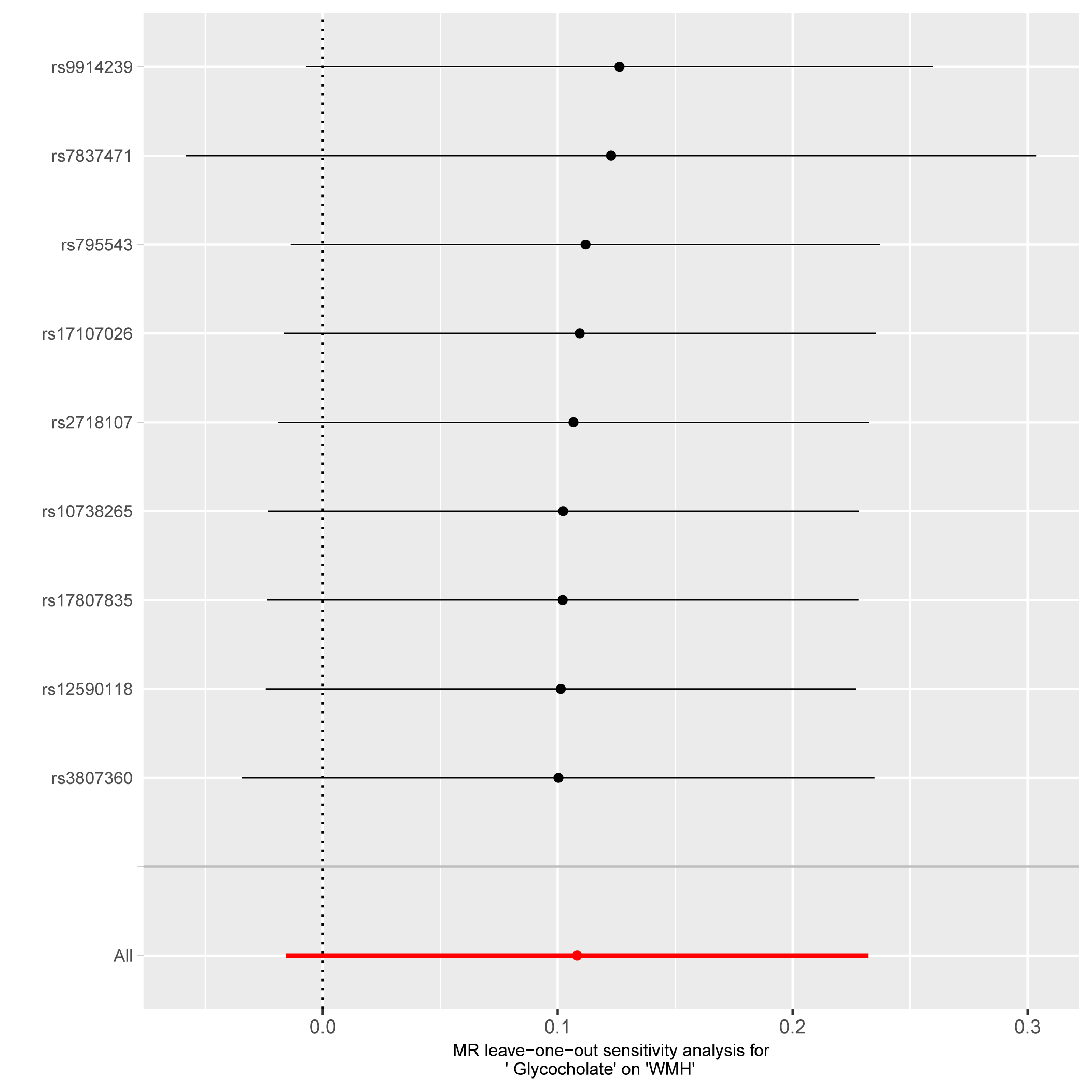

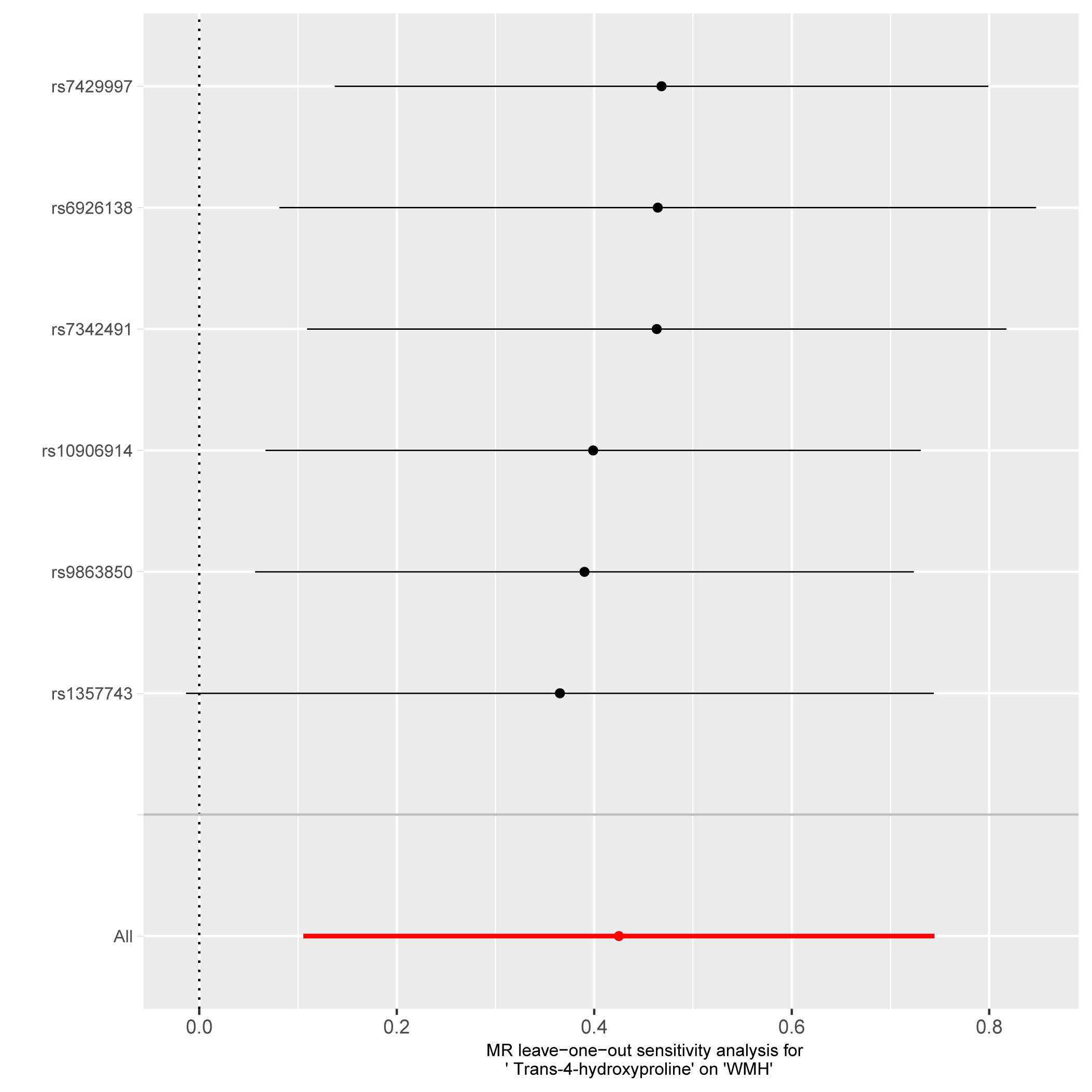

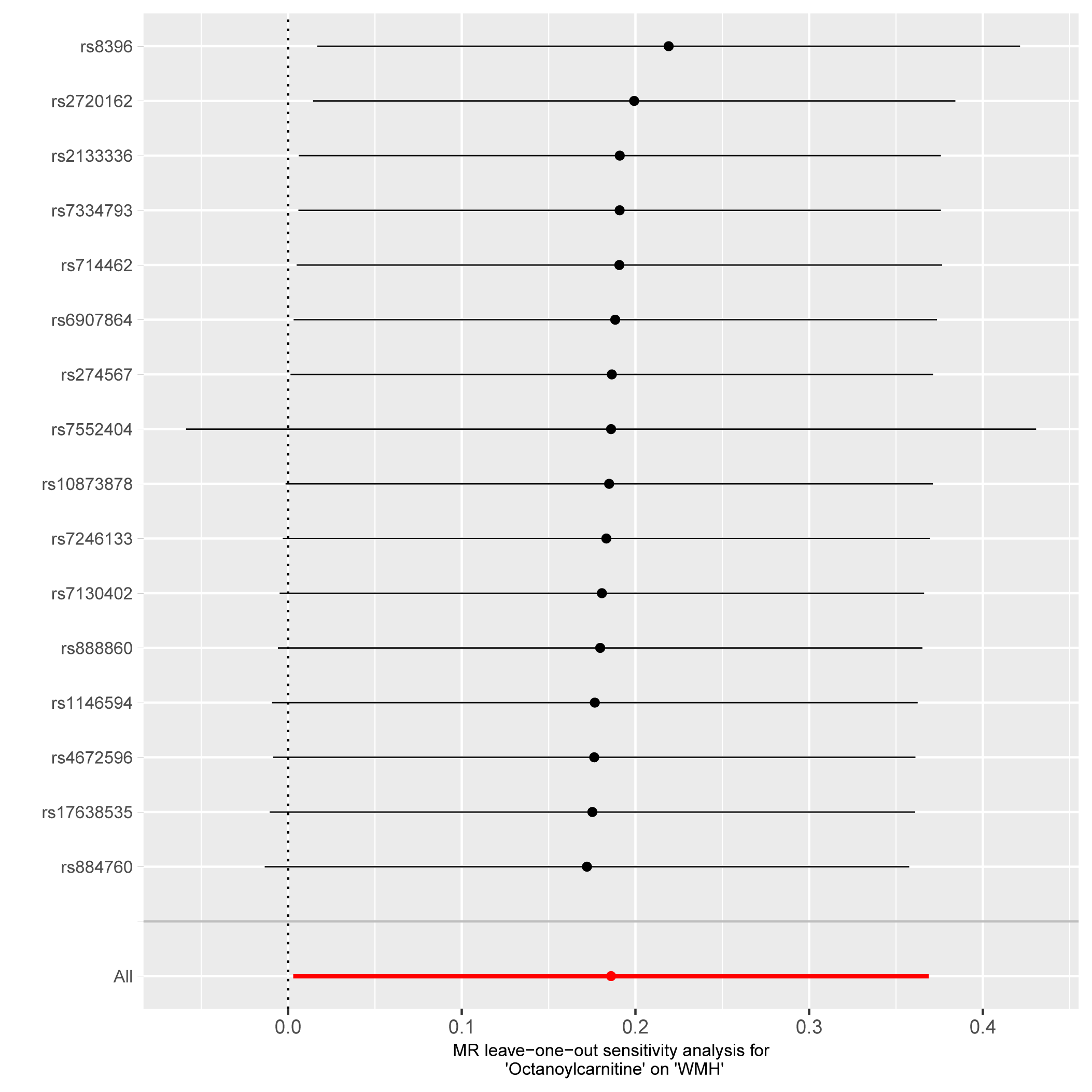

Supplement: Supplementary file 2 — Additional file 2: The result for leave-one-out analysis. [file 12967_2023_4677_MOESM2_ESM.docx]
